# Identity Matters for Well-Being: The Longitudinal Associations Between Identity Processes and Well-Being in Adolescents with Different Cultural Backgrounds

**DOI:** 10.1007/s10964-023-01901-8

**Published:** 2023-11-08

**Authors:** Francesca De Lise, Koen Luyckx, Elisabetta Crocetti

**Affiliations:** 1 Department of Psychology, Alma Mater Studiorum University of Bologna, Bologna, Italy; 2 Faculty of Psychology and Educational Sciences, KU Leuven, Leuven, Belgium

**Keywords:** Identity Processes, Well-Being, Adolescence, Migrant Background, Longitudinal

## Abstract

Adolescents’ identity processes and their levels of well-being are likely to be intertwined. On the one hand, how adolescents cope with the core developmental task of forming their identity has important implications for their well-being. On the other hand, experiencing a condition of well-being can help adolescents consolidate their identity. This longitudinal study adopted a multidimensional and culturally sensitive perspective to unravel how identity processes (i.e., commitment, in-depth exploration, and reconsideration of commitment) in two domains (i.e., educational and interpersonal identity) were developmentally related to multiple indicators of positive well-being (i.e., physical health, subjective, psychological, and social well-being) in adolescents with different cultural backgrounds. Participants were 1396 adolescents (*M*_age_ = 15.73, SD_age_ = 1.23, 49.93% females; 20.89% with a migrant background) who completed questionnaires at four-time points across one year. Results of cross-lagged models confirmed the positive reciprocal associations between identity commitment and well-being in all its facets. The nuanced picture of associations of in-depth exploration and reconsideration of commitment with multiple dimensions of well-being highlighted the importance of adopting a multi-dimensional perspective on well-being and a domain-specific approach to identity. Multigroup analyses indicated that the associations between identity commitment in the educational domain and well-being are relevant, especially for adolescents with a migrant background. Overall, this study highlights the centrality of identity processes for adolescents’ adaptation and points to a dynamic loop of reciprocal influences at the core of youth positive development.

## Introduction

Adolescents face the critical task of establishing a clear identity (Erikson, 1968) by exploring alternatives and making meaningful commitments in developmentally relevant domains, such as educational and interpersonal ones (Albarello et al., 2018). The extent to which adolescents develop a clear sense of themselves is closely related to their psychosocial functioning and well-being (Karaś & Cieciuch, 2018). Extensive research has focused on how identity relates to maladjustment, unraveling how identity processes are associated with internalizing (e.g., anxiety and depression; Crocetti, 2018; Crocetti et al., 2009) and externalizing (e.g., impulsivity, delinquency; Hatano et al., 2016; Mercer et al., 2017) problem behaviors. In line with the positive psychology movement (Seligman & Csikszentmihalyi, 2000), increasing attention has been paid also to how identity is related to positive well-being, such as to indicators of subjective (Diener et al., 1999), psychological (Ryff, 1989), and social well-being (Keyes, 2005). However, a still unexplored aspect concerns the interplay between identity and positive physical health perception. Another aspect that needs to be tackled is if, in a dynamic loop, experiencing well-being can help adolescents in developing their identity. Furthermore, the task of forming a stable identity might be particularly challenging for adolescents with a migrant background (individuals born or with at least one parent born outside the destination country; European Commission, 2023) who have to face, in addition to the developmental task, the acculturative one of integrating their culture of origin with the host one (Crocetti et al., 2023; Motti-Stefanidi, 2019). In line with this reasoning, this study, adopting a multi-dimensional domain-specific perspective on both identity (Crocetti et al., 2023) and well-being, aims to tackle the complex interplay between identity processes in developmentally-relevant domains and multiple dimensions of well-being in both adolescents from the majority group and their peers with a migrant background.

### Identity in Adolescence

Throughout life, individuals feel the urge to define themselves (Erikson, 1964) and to answer the question, “Who am I?”. In this vein, identity is a multi-dimensional construct that refers to a subjective sense of continuity across times and contexts (Meeus, 2023). Identity processes continue to evolve and change throughout the lifespan and are particularly central during adolescence, when individuals undergo multiple changes physically, psychologically, and socially (Crocetti, 2017, 2018). Thus, identity can be seen as a product of individuals’ continuous adjustment and regulation in different moments and contexts.

Several process models (for an overview, see Schwartz et al., 2011) have been conceptualized to explain how individuals develop their identity. These dynamic processes of identity definition and re-definition can be effectively captured by dual-cycle identity models (for a review, see Meeus, 2011). In this vein, the three-factor model represents a parsimonious approach to examining how individuals form and change their identity (Crocetti et al., 2008). This model focuses on the interplay of three processes: *commitment*, which refers to the choices that adolescents have made about various developmental domains and the self-confidence stemming from these choices; *in-depth exploration*, mainly the extent to which adolescents actively explore the commitments they have already made by reflecting on their choices and talking about them with others; and *reconsideration of commitment*, which refers to comparing current commitments with possible alternative and more satisfactory ones.

These processes are intertwined within two iterative cycles (Crocetti, 2017; Meeus, 2018). The cycle of identity formation (Cycle 1) results from the interplay between commitment and reconsideration of commitment processes. Individuals in this cycle revise their identity commitments in light of possibly more appealing alternatives. The maintenance cycle (Cycle 2) results from the interplay between commitment and in-depth exploration, whereby youth examine their current commitments and validate the choices that align with their goals. When these choices no longer harmonize well with their sense of self, individuals can return to the first identity formation cycle (Crocetti, 2018). These iterative cycles illustrate how individuals form, evaluate, and revise their identity over time.

These cycles operate in multiple life domains (Klimstra et al., 2016). The educational and interpersonal domains are particularly relevant for individuals’ meaningful life choices during adolescence. Adopting a domain-specific approach allows for differentiating between relatively more closed and more open domains (Meeus et al., 1999). On the one hand, educational identity is a relatively “closed” domain since the school system’s rules and norms may limit adolescents’ possibility to explore alternative paths and change their school track (Albarello et al., 2018). On the other hand, interpersonal identity can be considered a more “open” domain, where adolescents can explore a relatively wide range of alternatives by engaging in various groups and experiencing intimate relationships with their friends (Klimstra et al., 2010). Therefore, educational identity appears to be less changeable than interpersonal identity because of environmental constraints (Hatano et al., 2020). Hence, it is helpful to examine different domains separately, as adolescents with a strong sense of identity in one domain do not necessarily have a similar certainty in other domains, and each domain may contribute differently to their well-being (Crocetti et al., 2012).

### The Multiple Sides of Well-Being

The World Health Organization has conceptualized health as a general state of physical, psychological, and social well-being, not merely as the absence of disease or infirmity. The multidimensionality of well-being can be traced back to two philosophical conceptualizations, referring to the hedonic and eudaimonic approaches (Ryan & Deci, 2001). Hedonic well-being, on the one hand, focuses on the pursuit of pleasure and the avoidance of pain in various life domains, with higher levels of well-being characterized by increased life satisfaction and a predominance of positive affect (Diener et al., 1999). Eudaimonic well-being, on the other hand, centers around the notion of human flourishing, emphasizing the development of individuals’ true potential. The realization of these potentials is associated with higher psychological well-being (Ryff, 1989, 2014). Moreover, the social context is explicitly incorporated into the dynamics of well-being as an attempt to capture individual functioning within society (Keyes, 1998).

In this vein, it is possible to identify different dimensions of well-being. Specifically, *subjective well-being*, which may be regarded as an aspect of hedonic well-being, refers to individuals’ assessment of their own lives, taking into account overall life satisfaction, a sense of fulfillment, and the prevalence of positive over negative emotions (Diener et al., 1999). *Psychological well-being*, a eudaimonic facet of well-being, refers to the extent to which individuals see themselves positively, feel autonomous and in control of their lives, and are aware of their limitations and the possibility of realizing their potential (Ryff, 2014). *Social well-being* refers to the extent to which individuals feel that they are part of and can contribute to the society in which they are embedded (Keyes, 2005). To have a wider conceptualization of individuals’ state of well-being, it is necessary to consider also their perception of their physical health, which concerns one’s feeling of being in good health. This feeling is mainly due, during adolescence, to the interaction between early childhood development and specific biological and social changes that accompany puberty (Sawyer et al., 2012), and is affected by the presence or absence of health-related risk and protective factors (e.g., maintaining a balanced and nutritious diet, refraining from or limiting the use of alcohol and drugs, restorative and high-quality sleep).

In line with this multidimensional conceptualization (Bornstein et al., 2003), and acknowledging their distinct theoretical foundations, this study will examine four well-being dimensions separately. These dimensions include physical health perception, subjective well-being, psychological well-being, and social well-being. Notably, each of these well-being dimensions is likely to influence and be influenced by how adolescents confront their primary developmental task: forming and solidifying their own identity.

### The Link Between Identity and Well-Being

Emphasizing the significance of identity in the lives of adolescents, research has shown that successful coping with the identity task is intertwined with multiple well-being outcomes across different cultures (Branje, 2022). Studies have consistently revealed that adolescents who possess stable and firm identity commitments consistently report better psychosocial adjustment levels over time compared to their peers who struggle with ongoing identity uncertainty (Branje et al., 2021; Crocetti et al., 2023; Meeus, 2011). On the other side, previous studies have highlighted that psychosocial problems (i.e., depression, anxiety, Crocetti et al., 2009; Hatano et al., 2018) can hinder identity formation and consolidation, leading to the suggestion that identity and well-being may reinforce each other in a positive loop.

#### Does identity matter for well-being?

According to Erikson’s (1968) theory, identity fulfills a self-regulatory function: a clear sense of identity enables individuals to navigate significant life choices and provides them with a sense of control and a connection between the present, past, and future (Adams & Marshall, 1996). Thus, the more advanced adolescents are in consolidating their identity, the more they can benefit from its self-regulatory functions (Crocetti et al., 2013). Moving from these theoretical premises, identity processes may affect individuals’ adjustment and well-being.

So far, there is consistent evidence of the associations of identity processes with various correlates of well-being. However, most research focused primarily on indicators of internalizing and externalizing problem behaviors. Specifically, commitment was found to be negatively associated with internalizing problems, such as anxiety and depression (Crocetti et al., 2013; Schwartz et al., 2012), while in-depth exploration and reconsideration of commitment were positively linked to them. Finally, higher reconsideration was found to be associated with more self-reported delinquency (Crocetti et al., 2008; Mercer et al., 2017) and higher externalizing problems (Hatano et al., 2016).

In line with the positive psychology movement (Seligman & Csikszentmihalyi, 2000), research has started to increasingly account for the associations between identity processes and positive well-being outcomes (e.g., Bogaerts et al., 2023; Hatano et al., 2020). Commitment has been identified as an essential asset providing a sense of security and stability that enhances life satisfaction (Dimitrova et al., 2018; Rokvic et al., 2022) and psychological and social well-being (Karaś et al., 2015). In-depth exploration might be considered a double-edged sword since it has been found to be related to both negative (i.e., internalizing symptoms; Hatano et al., 2016) and positive (i.e., curiosity, openness to experiences, satisfaction with life, and social well-being; Karaś et al., 2015) aspects of well-being. Reconsideration of commitment was confirmed to be a troublesome aspect of identity formation, which may lead to decreased well-being (Karaś & Cieciuch, 2018).

#### Can well-being help identity formation processes?

While having a clear sense of identity can lead to better well-being, certain physical and mental health issues can hinder the process of forming a strong identity (Hatano et al., 2018; Meeus, 2023). Conversely, it is likely that the more adolescents feel good about themselves, their health, and their involvement in the society, the more confident they would feel in the process of validating and confirming meaningful choices to build a solid identity. Therefore, well-being states might function as protective factors to enhance identity formation processes.

Research has increasingly focused on how health and well-being can affect identity formation in different life domains. A few studies have shown that adolescents who experience somatic symptoms generally face more obstacles in actively exploring identity alternatives and making meaningful commitments (Raemen et al., 2023; Vankerckhoven et al., 2023). Moreover, psychosocial problems (i.e., feeling depressed, left out, and engaging in delinquent behavior), and anxiety may hamper adolescents’ identity opportunities (Crocetti et al., 2009; Hatano et al., 2018).

Moving from these premises, it is reasonable to assume that the relationship between identity and well-being might be bidirectional. Nevertheless, the available literature on the topic is still fragmented, and it is missing a comprehensive overview of these associations. While most studies focused on physical and mental illness as potential risk factors, there is a lack of research focused on investigating whether positive well-being can be a protective factor, enhancing identity consolidation. Therefore, the current longitudinal study aims to fill this gap, providing a nuanced understanding of the interplay of identity processes in different domains and different facets of well-being over time, by also taking a culturally sensitive approach.

### The Experience of Adolescents with a Migrant Background

If it is crucial to shed light on the associations between identity processes and well-being during adolescence, it is of utmost importance to investigate which factors might moderate these relations in order to clarify for whom they could be stronger or weaker. Previous studies highlighted that even though identity is a fundamental asset for individuals’ well-being, there are some differences in identity processes between cultural contexts (Crocetti et al., 2012). Specifically, the protective or detrimental role played by identity processes such as commitment and reconsideration of commitment on individuals’ well-being has been proven to change based on cultural and social diversity. For instance, in countries where adolescents perceive a wider array of alternatives (i.e., Italy, the Netherlands, China), commitment appeared to be a more decisive protective factor for anxiety symptoms. In contrast, in countries in which adolescents may perceive to have relatively lower possibilities (i.e., Kenya, Bulgaria, Philippines), reconsideration of commitment had a more substantial negative impact on adolescents’ anxiety (Crocetti et al., 2015).

In addition to cultural differences across various national contexts, societies are becoming increasingly more ethnically and culturally diverse thanks to migration processes (Bagci & Rutland, 2019). In the context of multicultural societies, the task of identity formation and consolidation can be more difficult for adolescents belonging to ethnic minorities due to the need to integrate different sets of values and norms from different cultures (Mastrotheodoros et al., 2021; Nguyen & Benet-Martínez, 2013; Phalet & Schönpflug, 2001). As such, these youth might develop both ethnic and national identities (Berry et al., 2006). While some adolescents can successfully develop a dual identity in which both ethnic and national identity are equally relevant, others can be more ethnic-oriented, national-oriented, or experience a marginalized identity, namely with low ethnic and national identity (Karataş et al., 2023; Mancini & Bottura, 2014).

Studies conducted using the three-factor identity model have consistently shown that ethnic minority adolescents often encounter more challenges when it comes to defining their identity across various domains, including education and relationships (e.g., Crocetti et al., 2008, 2011). These can, in turn, affect their adjustment and well-being. In light of these considerations, it appears of utmost importance to consider the cultural background as a variable that can weigh in determining the impact of identity processes on well-being and vice-versa. The current study sought to expand knowledge about this topic by investigating whether adolescents’ migrant background might be a moderating factor in the associations between identity processes in educational and interpersonal domains and youth’s well-being.

## Current Study

Extensive research has highlighted reciprocal associations between identity processes and well-being in adolescence. Although recent research has devoted increasing attention to the positive side of well-being, it has neglected to account for its multifaceted nature, leaving the longitudinal interplay of identity processes in different life domains (e.g., educational and interpersonal) and multiple dimensions of well-being (i.e., physical health, subjective, psychological, and social well-being) largely unexplored. Moreover, the role that having a migrant background has in affecting these relations has been poorly considered. The current study aimed to advance the understanding of these associations by focusing on two main aspects. First, it sought to consider how identity processes (i.e., commitment, in-depth exploration, and reconsideration of commitment) in two different domains (i.e., educational and interpersonal) are intertwined with multiple well-being dimensions (i.e., physical health perception, subjective, psychological, and social well-being), considered separately. Second, within a culturally sensitive perspective, associations were modeled considering whether effects could be moderated by adolescents’ migrant background. Therefore, adopting a multi-dimensional approach can provide a comprehensive picture of how identity processes in different domains and multiple facets of well-being are related in adolescents with different cultural backgrounds.

## Methods

### Participants

Participants of this study are drawn from the ongoing longitudinal ERC‐Consolidator project IDENTITIES “Managing identities in diverse societies: A developmental intergroup perspective with adolescents”. A sample of 1,396 adolescents (at baseline: *M*_age_ = 15.73, SD_age_ = 1.23, 49.93% females) attending the 1st (49.78%) and 3rd (50.22%) year of several high schools located in the Northern part of Italy (i.e., Emilia-Romagna region) were included. Specifically, students were enrolled in different types of high schools (i.e., lyceum 41.75%, technical 33.36%, and professional institutes 24.89%). The sample involved both Italian adolescents (79.01%) and adolescents with a migrant background (20.89%; i.e., those who were born in another country or with at least one of their parents born in another country). Among them, 37.98% had a European background, with Albanians and Romanians as the most highly represented groups. The origins of other participants were from Asia (29.11%, mostly China and India), Africa (24.05%), North, Central, and South America (7.59%), and Australia (1.27%). Overall, these numbers reflect the socio-demographic characteristics of the ethnic minorities living in the Italian context. Specifically, according to national statistics (ISTAT, 2020), most migrants living in Italy are from Eastern European countries, such as Romania (21.5%) and Albania (8.3%), followed by those of African (e.g., Moroccans, who represent the 8.3%) and Asian (e.g., Chinese, who represent almost 6% of the ethnic minority population) origins. Regarding parents’ educational level, most of the adolescents’ mothers (47.37%) had a medium educational level (i.e., high school diploma), while the others (31.85%) had a high (i.e., university degree or higher) or a low (i.e., up to middle school diploma) educational level (20.78%). As for fathers, most of them (46.97%) had a medium educational level, followed by those with low (29.10%) and high (23.93%) educational levels.

Among the total sample, 44.41% of adolescents participated in all four assessments, while 18.12%, 17.55%, and 19.91% participated in three, two, and one assessment, respectively. More specifically, participants were 1,156 at T1, 1,063 at T2, 928 at T3, and 860 at T4. Within each assessment, the completion rate of the questionnaires was high, ranging from 77.36% at T1 to 56.02% at T4. To gather a better understanding of the sample attrition, additional analyses were completed to ensure that the attrition was not related to specific variables by comparing adolescents who participated in all four assessments with those who attended only three, two, or one assessment. Results (available in Supplementary Materials S1) revealed that the four groups were largely comparable. The Little’s (1988) Missing Completely at Random (MCAR) test yielded a normed χ^2^ (χ^2^/df = 3875.16/3135) of 1.24, indicating that data were likely missing completely at random. Therefore, the total sample of 1,396 participants was included in the analyses, and missing data were handled with the Full Information Maximum Likelihood (FIML) option available in M*plus* (Kelloway, 2015).

### Procedure

The present study was approved by the Ethics Committee of Alma Mater Studiorum University of Bologna (Italy) as part of the ERC‐Consolidator project IDENTITIES “Managing identities in diverse societies: A developmental intergroup perspective with adolescents”. Upon their approval, the study was presented to students and their parents, who received detailed oral and written information. Active consent from parents was obtained prior to their children’s participation. Active consent was also obtained from adolescents of age while their underage peers provided their assent to participate in the project. Participation in the study was voluntary, and students were informed they could withdraw their consent at any time.

The IDENTITIES project started in 2022 and includes multiple assessments. For the present study, adolescents’ data collected amid one school year (i.e., January/February 2022, T1), at the end of the same school year (i.e., April/May 2022, T2), and at the beginning (i.e., September/October 2022, T3), and in the midst (i.e., January/February 2023, T4) of the following school year were used. At each wave, adolescents completed an online questionnaire during class hours. They were required to create a personal code to ensure confidentiality and to be able to link their answers over time while protecting their anonymity.

### Measures

#### Demographics

Participants’ socio-demographic informations (e.g., age, biological sex, parents’ educational level) were collected at the beginning of the study.

#### Identity processes in educational and interpersonal domains

Commitment, in-depth exploration, and reconsideration of commitment in the educational and interpersonal domains were measured using the Utrecht-Management of Identity Commitments Scale (U-MICS, Crocetti et al., 2008; Italian validation by Crocetti et al., 2010). The instrument consists of 13 items scored on a 5-point Likert-type rating scale, ranging from 1 (*completely false*) to 5 (*completely true*), reported for the two domains. Sample items include: “My education/The relationship with my best friend gives me certainty in life” (commitment; 5 items), “I think a lot about my education/the relationship with my best friend” (in-depth exploration; 5 items), and “I often think it would be better to try to find a different education/a new best friend” (reconsideration of commitment; 3 items). Cronbach’s Alphas across the four waves ranged from 0.89 to 0.93 for commitment, from 0.74 to 0.79 for in-depth exploration, and from 0.77 to 0.82 for reconsideration of commitment in the educational domain; from 0.88 to 0.93 for commitment, from 0.71 to 0.79 for in-depth exploration, and from 0.86 to 0.92 for reconsideration of commitment, in the interpersonal domain.

#### Physical health perception

Adolescents’ physical health perception was assessed using the “General Health” subscale from the Short Form-36 Health Survey (SF-36; Ware & Gandek, 1994; for the Italian version, see Apolone & Mosconi, 1998). The instrument consists of five items of which one (i.e., “In general, would you say your health is”) scored on a 5-point Likert-type rating scale, ranging from 1 (*poor*) to 5 (*excellent*) and the remaining four items (e.g., “My health is excellent”) scored on a 5-point Likert-type rating scale, ranging from 1 (*completely false*) to 5 (*completely true*). Cronbach’s Alphas ranged from 0.74 to 0.78 across the four waves.

#### Subjective, psychological, and social well-being

Well-being was assessed employing the Mental Health Continuum –Short Form (MHC-SF; Keyes, 2005; Italian validation by Petrillo et al. 2015), which measures three sub-components of well-being, that is subjective, psychological, and social well-being. This scale consists of 14 items referred to the last 4 months. Ratings were expressed on a 6-point Likert-type scale from 1 (*never*) to 6 (*every day*). Sample items are the following: “How often did you feel happy?” (subjective well-being; 3 items); “How often did you feel good at managing the responsibilities of your daily life?” (psychological well-being; 6 items), and “How often did you feel that you had something important to contribute to society?” (social well-being; 5 items). Cronbach’s Alphas across the four waves ranged from 0.81 to 0.86 for subjective well-being, from 0.81 to 0.89 for psychological well-being, and from 0.75 to 0.85 for social well-being.

## Results

### Preliminary Analyses

Descriptive analyses were computed using IBM SPSS Version 26.0 for Windows. Means, standard deviations, and correlations among study variables are reported in Table S2a–S2b of the Supplemental Materials. All the remaining analyses were conducted in M*plus* 8.10 (Muthén & Muthén, 2017), using the Maximum Likelihood Robust (MLR) estimator (Satorra & Bentler, 2001). As a preliminary step, longitudinal measurement invariance was examined separately for all study variables (see Table S3 of the Supplemental Materials). Since metric invariance was established for all constructs, observed variables could be used to test the main hypotheses. Data, analyses codes, and outputs can be retrieved from https://osf.io/jmxsp/.

### Cross-Lagged Associations of Identity and Well-Being

The current study sought to disentangle reciprocal longitudinal associations between identity processes (i.e., commitment, in-depth exploration, and reconsideration of commitment) in educational and interpersonal domains, physical health perception, and subjective, psychological, and social well-being. To this end, four cross-lagged panel models (distinguished for each dimension of well-being) with observed variables were tested, controlling for (a) stability or autoregressive paths (i.e., T1 → T2, T2 → T3, T3 → T4), (b) within-time correlations among all variables (i.e., correlations among variables at T1, and correlated changes at T2, T3, and T4), and (c) the effects of participants’ sex (0 = boys, 1 = girls) and age.

To identify the most parsimonious model of reciprocal associations, at first unconstrained models (M1) were tested. All four models tested showed a very good fit, based on a combination of the following indices (Byrne, 2012): the Comparative Fit Index (CFI) and the Tucker-Lewis Index (TLI), with values higher than 0.90 and 0.95 indicative of acceptable and very good fit, respectively; and the Root Mean Square Error of Approximation (RMSEA) and the Standardized Root Mean Residual (SRMR) with values below 0.08 and 0.05 indicative of an acceptable and very good fit, respectively. Additionally, the RMSEA’s 90% confidence interval’s upper bound lower than 0.10 indicates an acceptable model fit (Chen et al., 2008). Further, models (M2) with cross-lagged paths fixed to be equal across waves (i.e., T1 → T2 paths constrained to be equal to T2 → T3 and T3 → T4 paths) were tested and compared against the unconstrained ones, and models (M3) with fixed cross-lagged paths and fixed correlated changes (i.e., within-time correlations at T2, T3, and T4) were tested and compared against M2. Differences between models were identified if at least two of the following criteria were met: a Δχ_SB_^2^ significant at *p* < 0.05 (Satorra & Bentler, 2001), ΔCFI ≥ −0.010, and ΔRMSEA ≥ 0.015 (Chen, 2007). Results indicated that time-invariance of both cross-lagged paths and correlated changes could be established (see Table S4 of the Supplemental Materials) for all four models. Thus, the most parsimonious solutions (M3) were retained as the final ones.

**Fig. 1:**
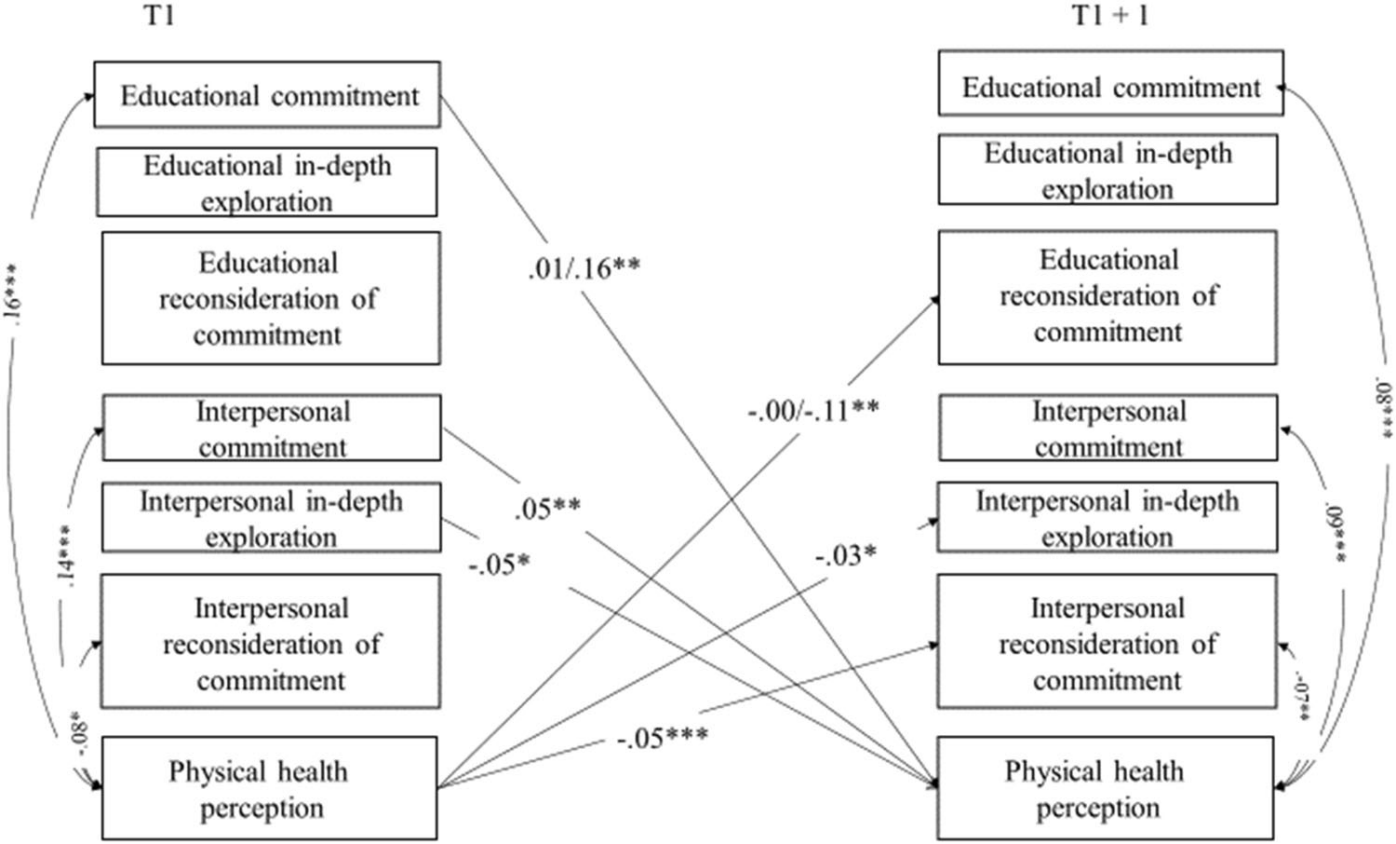
Significant standardized results of the identity and physical health perception cross-lagged panel model: Cross-lagged paths and correlations. For the sake of clarity, only significant cross-lagged paths and correlations linking identity processes to well-being are displayed. When multigroup analyses revealed a significant difference, two effects are reported on the same path/correlation (the first one regarding the majority group, and the second one regarding adolescents with a migrant background). Sex and age were included in the model as covariates. *p < 0.05; **p < 0.01; ***p < 0.001

**Fig. 2:**
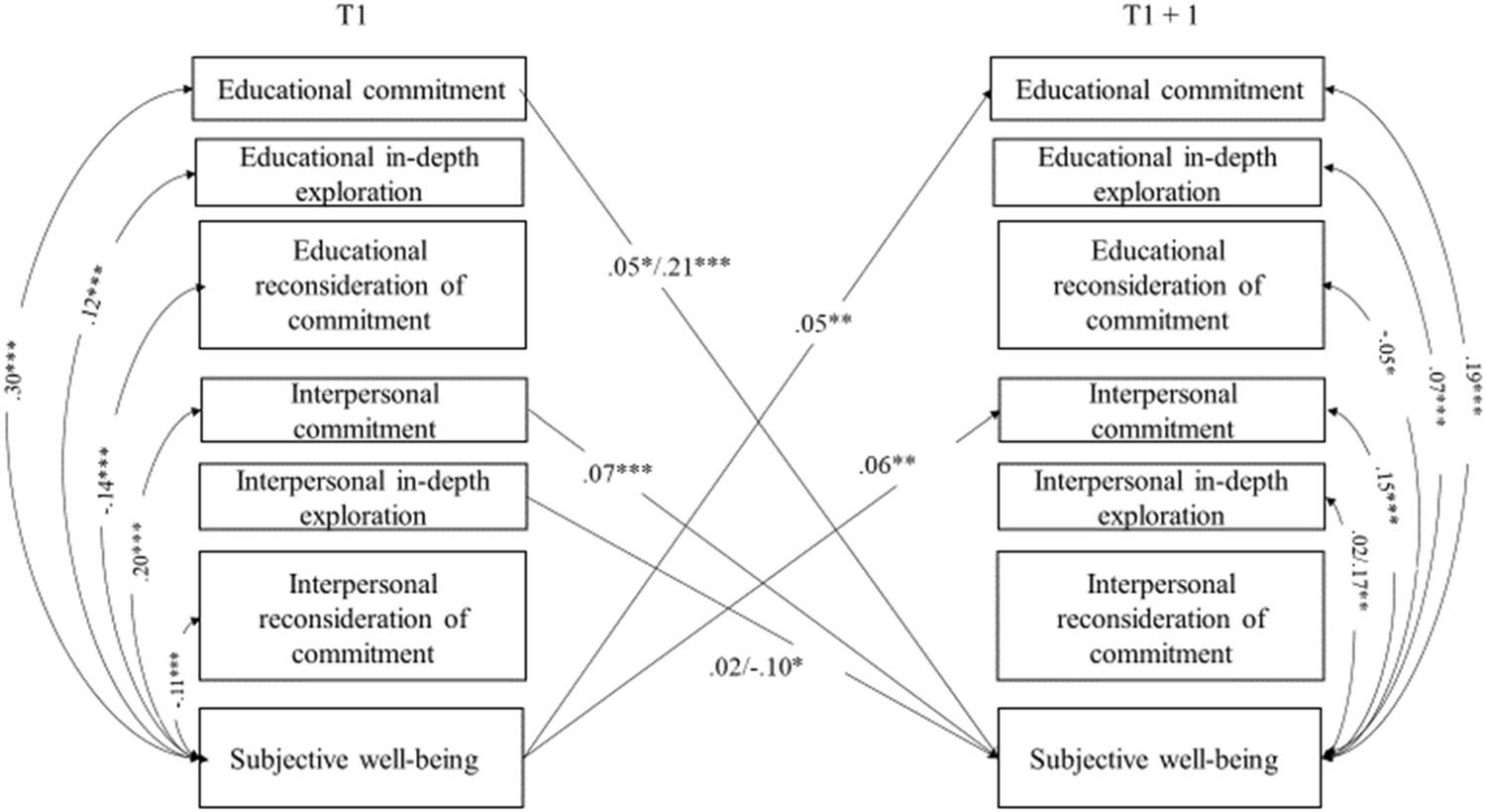
Significant standardized results of the identity and subjective well-being cross-lagged panel model: Cross-lagged paths and correlations. For the sake of clarity, only significant cross-lagged paths and correlations linking identity processes to well-being are displayed. When multigroup analyses revealed a significant difference, two effects are reported on the same path/correlation (the first one regarding the majority group, and the second one regarding adolescents with a migrant background). Sex and age were included in the model as covariates. *p < 0.05; **p < 0.01; ***p < 0.001

**Fig. 3:**
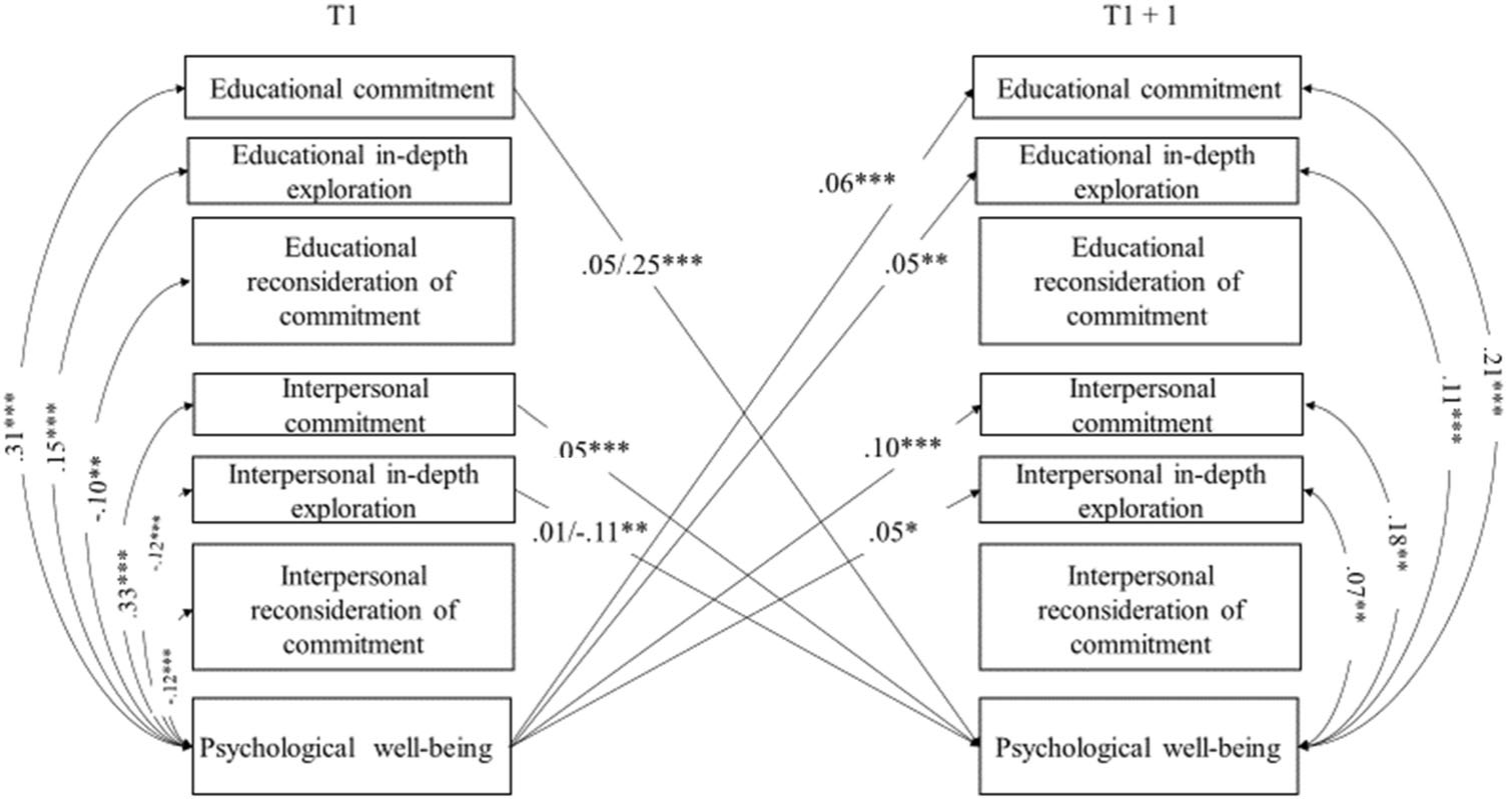
Significant standardized results of the identity and psychological well-being cross-lagged panel model: Cross-lagged paths and correlations. For the sake of clarity, only significant cross-lagged paths and correlations linking identity processes to well-being are displayed. When multigroup analyses revealed a significant difference, two effects are reported on the same path/correlation (the first one regarding the majority group, and the second one regarding adolescents with a migrant background). Sex and age were included in the model as covariates. *p < 0.05; **p < 0.01; ***p < 0.001

**Fig. 4:**
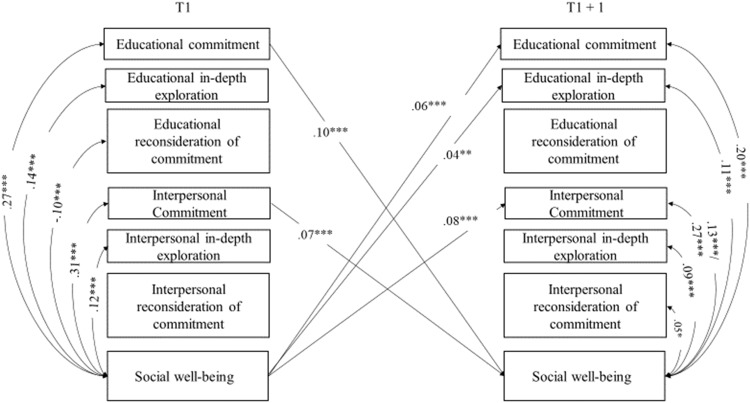
Significant standardized results of the identity and social well-being cross-lagged panel model: Cross-lagged paths and correlations. For the sake of clarity, only significant cross-lagged paths and correlations linking identity processes to well-being are displayed. When multigroup analyses revealed a significant difference, two effects are reported on the same path/correlation (the first one regarding the majority group, and the second one regarding adolescents with a migrant background). Sex and age were included in the model as covariates. *p < 0.05; **p < 0.01; ***p < 0.001

Stability paths are reported in Table 1. As can be seen, all variables reported high stability over time. Significant cross-lagged paths, within-time correlations (T1), and correlated changes (T2, T3, and T4) between identity processes and well-being are reported in Figs. 1–4 and discussed below. All models controlled for adolescents’ sex and age, which were included as covariates and left unconstrained, allowing them to exert potentially different effects on identity processes and well-being over time. Tables with all paths and correlations are available in Supplemental Materials (Table S5a, S5b, S5c). To highlight possible significant differences in paths and correlations between adolescents from the majority group and those with a migrant background, multigroup analyses were conducted. Associations were modelled separately for the two groups and the Wald-based statistic test was used to perform pairwise comparisons between parameters.

**Table 1:** Standardized results of the cross-lagged panel model with covariates: Stability paths

	Model 1 - Identity and physical health perception			Model 2 - Identity and subjective well-being			Model 3 - Identity and psychological well-being			Model 4 - Identity and social well-being		
Stability paths	T1 → T2	T2 → T3	T3 → T4	T1 → T2	T2 → T3	T3 → T4	T1 → T2	T2 → T3	T3 → T4	T1 → T2	T2 → T3	T3 → T4
Educational commitment	0.57^***^	0.60^***^	0.61^***^	0.56^***^	0.60^***^	0.60^***^	0.55^***^	0.60^***^	0.60^***^	0.55^***^	0.59^***^	0.59^***^
Educational in-depth exploration	0.50^***^	0.53^***^	0.50^***^	0.50^***^	0.53^***^	0.50^***^	0.50^***^	0.53^***^	0.50^***^	0.50^***^	0.53^***^	0.50^***^
Educational reconsideration of commitment	0.53^***^	0.53^***^	0.57^***^	0.54^***^	0.53^***^	0.57^***^	0.53^***^	0.53^***^	0.57^***^	0.53^***^	0.53^***^	0.54^***^
Interpersonal commitment	0.52^***^	0.54^***^	0.56^***^	0.51^***^	0.52^***^	0.55^***^	0.49^***^	0.51^***^	0.53^***^	0.50^***^	0.51^***^	0.54^***^
Interpersonal in-depth exploration	0.51^***^	0.51^***^	0.52^***^	0.51^***^	0.52^***^	0.52^***^	0.51^***^	0.52^***^	0.53^***^	0.51^***^	0.52^***^	0.53^***^
Interpersonal reconsideration of commitment	0.46^***^	0.47^***^	0.53^***^	0.46^***^	0.47^***^	0.54^***^	0.46^***^	0.47^***^	0.54^***^	0.46^***^	0.47^***^	0.54^***^
Physical health perception	0.62^***^	0.65^***^	0.67^***^									
Subjective well-being				0.56^***^	0.59^***^	0.56^***^						
Psychological well-being							0.58^***^	0.53^***^	0.54^***^			
Social well-being										0.50^***^	0.51^***^	0.52^***^

T = Time

^***^p < 0.001

#### The interplay of identity processes and physical health perception

As cross-lagged paths displayed in Fig. 1 indicate, commitment in the interpersonal domain was positively associated with physical health perception over time. Moreover, there was a reciprocal negative association between in-depth exploration in the interpersonal domain and physical health. This latter dimension was associated with a lower reconsideration of commitment in both the educational and the interpersonal domains over time. Multigroup analyses revealed that these results were largely replicated except for the effect of educational commitment on physical health perception that was significantly stronger (Wald = 7.41; *p* = 0.006) in adolescents with a migrant background (*β* = 0.16; *p* = 0.002) than in adolescents from the majority group (*β* = 0.01; *p* = 0.618), and for the effect of physical health perception on educational reconsideration of commitment that was also stronger (Wald = 4.45; *p* = 0.035) for adolescents with a migrant background (*β* = −0.11; *p* = 0.017), compared with the majority group (*β* = −0.00; *p* = 0.922).

Concerning significant within-time correlations at baseline, commitment in both domains was positively associated with physical health perception, while reconsideration of commitment in the interpersonal domain was negatively associated with it. The same results were replicated in the correlated changes. Multigroup analyses did not reveal any significant difference between them.

#### The interplay of identity processes and subjective well-being

As shown in Fig. 2, commitment in both educational and interpersonal domains was positively related to subjective well-being over time. These cross-lagged associations were reciprocal, with higher levels of subjective well-being associated with higher levels of commitment in both domains over time. Consistent with the previous findings, multigroup analyses revealed that the effect of educational commitment on subjective well-being was stronger (Wald=6.24; *p* = 0.012) for the group with a migrant background (*β* = 0.21; *p* = 0.001) compared to the majority group (*β* = 0.05; *p* = 0.045). Additionally, a negative effect of interpersonal in-depth exploration on subjective well-being was found only for adolescents with a migrant background (Wald=5.46 *p* = 0.02; *β* = −0.01; *p* = 0.023), compared with the majority group (*β* = 0.02; *p* = 0.481).

As for within-time correlations at baseline, subjective well-being was positively associated with commitment and negatively associated with reconsideration of commitment, in both domains. Educational in-depth exploration was positively associated with this dimension of well-being. These results were replicated in the correlated changes, together with a positive association between interpersonal in-depth exploration and subjective well-being Further, multigroup analyses revealed a significant negative correlated change between interpersonal in-depth exploration and subjective well-being only for the minority group.

#### The interplay of identity processes and psychological well-being

As shown in Fig. 3, cross-lagged associations between identity processes and psychological well-being showed a reciprocal positive association with educational and interpersonal commitment. Furthermore, psychological well-being was also associated with higher in-depth exploration in both domains. Regarding multigroup analyses, results showed a significant difference between the majority and the minority group (Wald=9.15; *p* = 0.002), with a stronger effect of educational commitment on psychological well-being for the latter group (*β* = 0.25; *p* = 0.000), compared to the former group (*β* = 0.05; *p* = 0.035). A significant difference (Wald=5.53; *p* = 0.019) was found in the negative effect of in-depth exploration in the interpersonal domain on psychological well-being, which was more robust for the minority group (*β* = −0.11; *p* = 0.015) compared to the majority one (*β* = −0.01; *p* = 0.708).

As regards within-time correlations at baseline, consistent with the previous models, psychological well-being was positively associated with commitment and negatively associated with reconsideration of commitment in both domains. In-depth exploration was positively and negatively associated with this dimension of well-being for the educational and the interpersonal domains, respectively. Nevertheless, looking at correlated changes between commitment and in-depth exploration in both domains they were positively associated with psychological well-being. Correlated changes between reconsideration of commitment in both domains and psychological well-being turned out to be insignificant.

#### The interplay of identity processes and social well-being

Concerning cross-lagged associations with social well-being, displayed in Fig. 4, commitment in both educational and interpersonal domains was positively related to this well-being dimension over time. These associations were reciprocal, with higher levels of social well-being associated with higher levels of commitment in both domains over time. Social well-being was also positively related to later levels of educational in-depth exploration. Multigroup analyses did not reveal any differences between the minority and majority groups.

Within-time correlations at baseline revealed a positive association between social well-being and commitment and in-depth exploration in both domains. Conversely, a negative association was found between educational reconsideration of commitment and social well-being. These results were replicated in the correlated changes, except for the association between social well-being and educational reconsideration of commitment (which turned out to be insignificant). Nevertheless, there was a positive association in correlated changes between this well-being dimension and interpersonal reconsideration of commitment. Results of multigroup analyses showed a significant difference in the association between the correlated changes of commitment in the interpersonal domain and social well-being (Wald=5.68; *p* = 0.017), with a stronger relation for the adolescents with a migrant background (*β* = 0.27; *p* < 0.001), compared to ones from the majority group (*β* = 0.13; *p* < 0.001).

### Sensitivity Analyses

All model results described above controlled for the effects of sex and age (for detailed results see Table S5b of the Supplemental Materials). Results indicated that, regarding sex differences, girls reported higher levels of in-depth exploration in both domains at T2, whereas they displayed lower levels of reconsideration of commitment in the interpersonal and educational domains at T3 and T4, respectively. Boys reported higher levels of physical health perception at T3 and T4, and of subjective, psychological, and social well-being at T2, T3, and T4. Regarding participants’ age, younger students reported higher levels of educational commitment and in-depth exploration at T4 and T2, respectively. Conversely, older students displayed higher levels of interpersonal commitment at T2. Further, physical health perception was higher for younger students at T2. Thus, these results highlighted a few associations of sex and age with identity processes and well-being.

As ancillary sensitivity analyses, multigroup analyses were tested on the four cross-lagged models, to check whether sex and age, instead of being treated as covariates, as done in the main models discussed above, could moderate the results. To this end, the Wald-based statistics tests were conducted to test, one parameter at a time, the moderating role of sex and school year (i.e., first or third year at T1), which is the proxy for the two age groups examined in this study. Comparisons were done for cross-lagged paths (from identity to well-being and vice-versa), within-time correlations at baseline, and correlated changes, for a total of 24 tests per model. Detailed results can be retrieved in Supplemental Materials (Table S6). In total, sex was found to moderate only three associations across all models. The positive effect of social well-being on educational commitment over time, was significantly stronger for the boys, compared with the girls. Conversely, participants’ sex influenced the effect of interpersonal commitment on social well-being over time, which was significantly stronger for girls, compared with boys. Finally, the correlated change between physical health perception and educational in-depth exploration was different for boys and girls, with a positive association for the former group and a negative one for the latter.

Regarding participants’ age, this variable was found to moderate nine associations across all models. The effect of psychological well-being on educational reconsideration of commitment, and on commitment and in-depth exploration in the interpersonal domain, was more robust for older adolescents. Vice-versa, the effect of interpersonal in-depth exploration on this well-being dimension was found to be significantly stronger for younger participants. The effect of interpersonal in-depth exploration on social well-being was found to be stronger for younger students, whereas for the opposite direction, from social well-being to interpersonal in-depth exploration, the effect was more robust for older students. The association between educational reconsideration of commitment and subjective well-being over time was significantly stronger for older students, compared with younger students. Younger students presented a stronger correlation at baseline between interpersonal reconsideration of commitment and subjective well-being, as well as a stronger one with psychological well-being. Overall, except for these few paths, the results of the main models were largely replicated, highlighting the robustness of the findings.

## Discussion

Building a solid identity in different life domains is a pivotal task during adolescence, affecting individuals’ adjustment and well-being over time. However, adolescents’ psychosocial functioning can also play a role in the extent to which they feel safe to explore meaningful alternatives and can engage in identity formation processes. Extensive research has explored the relations occurring between identity and adjustment during adolescence and has increasingly focused on the positive side of well-being (i.e., positive physical health perception, subjective, psychological, and social well-being), considering the positive loop that the interplay of identity and well-being during adolescence might represent. The purpose of this study was to provide a comprehensive picture of the associations of identity processes and well-being in adolescence, adopting a domain-specific approach to identity and accounting for multiple dimensions of well-being. Moreover, the differences that may occur in these relations between adolescents from the majority group (i.e., Italian) and those with a migrant background (i.e., born or with at least one parent born in a different country), were also considered.

### The Positive Loop Between Identity and Well-Being

Previous research showed that a solid identity (i.e., characterized by high commitment, high in-depth exploration, and low reconsideration of commitment; Crocetti et al., 2008) is associated with better outcomes in terms of adjustment and mental health. Results of the present study advance this evidence, revealing a nuanced pattern of associations between identity processes in the educational and interpersonal domains, physical health perception, and subjective, psychological, and social well-being. Doing so, the present study contributes to a better understanding of the differences between identity domains (Klimstra et al., 2016) and focuses on the positive sides of well-being (Bornstein et al., 2003).

First of all, *commitment* in both domains appeared to be positively associated with all well-being dimensions over time. These findings highlight that this process represents a fundamental asset, not only to protect from disturbances (Raemen et al., 2022) but also to enhance positive feelings such as those accounted by well-being dimensions (Bogaerts et al., 2023). The underlying mechanism that can explain these results points to the role played by identity commitments, which give individuals a sense of direction and purpose and facilitate personal autonomy and effectiveness by providing standards for solving problems, making decisions, and interpreting self-relevant information (Berzonsky & Papini, 2015; Bosma & Kunnen, 2001). Moreover, commitment has been linked to a stable self-concept, emotional stability, and good relations with peers and parents, connected in turn to high levels of well-being (Karaś et al., 2015; Luyckx et al., 2011).

Importantly, results highlighted that all dimensions of well-being, except for physical health perception, were associated with higher levels of commitment in both domains over time. These results point to a positive loop between identity and well-being, that is the more adolescents feel in control of their life, that their life has meaning, and that they are involved in the society, the more confident they would feel in making meaningful choices in multiple life domains. The strong interplay between commitment and well-being dimensions was further corroborated by within-time correlations at baseline and correlated changes, which were consistently significant and highlighted the robustness of this “alliance” between commitment and well-being.

Further, *in-depth exploration* in the interpersonal domain was found to be reciprocally negatively associated with physical health perception over time. The negative reciprocal link between interpersonal in-depth exploration and physical health perception could be explained by considering that the interpersonal domain is strongly related to feeling social support from significant others (Hatano et al., 2020). The uncertainty regarding the relationship with their best friend can lead to negative feelings connected with stress and anxiety (Crocetti et al., 2009), making adolescence perceive their general health as poorer. These negative associations are in line with the idea that adolescents who display somatic symptoms are generally more impeded in actively exploring identity alternatives (Raemen et al., 2023). Conversely, higher levels of psychological and social well-being were associated with higher in-depth exploration in the educational domain. These results corroborate the idea that high levels of psychological well-being can function as a protective factor for adolescents to get engaged in the process of exploring in-depth their identity choices. This can be particularly evident in the educational domain because of its relatively more closed nature, which requires overcoming some contextual constraints to dive into a deep reflection (Becht et al., 2017). This relation was also confirmed by within-time correlations and correlated changes, where subjective, psychological, and social well-being were positively associated with in-depth exploration in the educational domain.

In this vein, this study further illuminates the complex role played by in-depth exploration, that has led to consider it as a double-edged sword (e.g., Crocetti, Jahromi, et al., 2012). On the one hand, in-depth exploration is beneficial at an individual, interpersonal, and collective level, being linked to adaptive personality dimensions such as extroversion, agreeableness and conscientiousness (Crocetti et al., 2008; Klimstra et al., 2012; Luyckx et al., 2014a, 2014b), warmer family and peer relationships (Crocetti et al., 2017; Kaniušonytė et al., 2019), higher social responsibility (Crocetti et al., 2012)), lower prejudice against minorities (Bobba et al., 2023), thus aspects that are fundamental to develop inclusive relations with diverse others. On the other hand, these positive aspects come with a certain cost for the individual, as in-depth exploration may also lead to distress and uncertainty about one’s choices (Crocetti et al., 2008; Karaś & Cieciuch, 2018). This nuanced picture highlights that the implications of in-depth exploration differ according to the level of analysis taken into account.

Finally, regarding *reconsideration of commitment*, physical health perception was associated with lower levels of this process in the interpersonal domain over time. In this way, adolescents who perceive that they are in good health, are less likely to reconsider their commitments regarding their interpersonal identity. This result confirms the importance of considering physical health perception as a unique asset when investigating adolescents’ well-being (Vankerckhoven et al., 2023), which can function as a protective factor for youth to form a solid identity.

This result was substantially replicated in the within-time correlations and correlated changes of the other models. Reconsideration of commitment in both domains was negatively associated with subjective and psychological well-being at baseline. Educational reconsideration of commitment was negatively associated with subjective well-being, whereas interpersonal reconsideration of commitment was positively associated with social well-being, in the correlated changes. This difference can be interpreted in light of the difference between the two dimensions of well-being, mainly considering the specificity of social well-being. It is likely that the more adolescents feel that they are part of the society in which they are embedded, the more they feel that they can revise their interpersonal commitment when they are no longer satisfied. This result reinforces the need to separately investigate different dimensions of well-being and to further explore their longitudinal association with identity formation.

Overall, these findings show how identity has a crucial role in promoting well-being during adolescence. Remarkably, the interplay of identity processes and well-being creates a positive loop in which a more solid identity is related to higher levels of adjustment over time and vice-versa. Even though the effect sizes of the paths were small, they can be considered meaningful in light of the stability of the variables, and of the significant bivariate correlations between them (Adachi & Willoughby, 2015). Importantly, these results highlight different paths depending on the identity domains, and the diverse dimensions of well-being considered.

### The Challenge of Integration: The Role of Different Cultural Backgrounds

One of the purposes of the current study was to take a culturally sensitive perspective to investigate whether having a migrant background might moderate the interplay of identity processes and well-being dimensions in adolescence. Interestingly, the results indicated that, albeit most patterns were replicated across groups, some differences regarding the cross-lagged association between identity processes in both the educational and interpersonal domains and dimensions of well-being emerged. Specifically, some paths resulted significantly stronger for youth with a migrant background than for those from the majority group.

Regarding the *educational domain*, the effect of commitment on physical health perception, subjective, and psychological well-being was significantly stronger for adolescents with a migrant background compared to the majority group. The negative association of physical health perception with reconsideration of commitment over time was significantly stronger for students with a migrant background. This link can be explained by considering the cultural differences that might occur between the groups. Adolescents with a migrant background need to navigate both the developmental and the acculturative tasks (Crocetti et al., 2023; Motti-Stefanidi, 2019), thus for them finding meaningful commitments is more difficult (Crocetti et al., 2011). This study shows that if solid commitments in the educational domain foster adolescents’ overall well-being in general, this association might be even stronger for youth with a migrant background who need to face more challenges in their adjustment process. These results call attention to the need to further explore the link between physical health perception and identity formation cycle in the educational domain. These findings also align with previous studies highlighting the importance of the school context in fostering integration (Bohman & Miklikowska, 2020) and minorities’ social class mobility (Platt, 2007).

Regarding the *interpersonal domain*, the association of in-depth exploration with both subjective and psychological well-being was significantly stronger for adolescents with a migrant background, compared with the majority group. A significant difference was found between the correlated changes in interpersonal commitment and those in social well-being. This result can be explained in light of the relational challenges to which the increasing multiculturality of nowadays society exposes adolescents (Karataş et al., 2023; Svensson & Syed, 2019). Specifically, individuals who belong to minority groups face the difficult task of trying to fit in, and in some cases struggling with the complex process of integrating some sides of the self that belong to their ethnic group of origin with some other sides that are part of the host culture (Mastrotheodoros et al., 2021). In this vein, in adolescents with a migrant background, the process of in-depth exploration concerning the relationship with their best friend can make them feel the actual possibility of going through alternative possibilities that in turn might support finding fulfilling commitments and, therefore, the achievement of a future solid identity that fosters adaptive functioning and well-being.

### Practical Implications

This study has noteworthy practical implications. First of all, since results highlight the positive loop between identity and well-being, it can be useful to plan evidence-based interventions, for instance, in schools, to foster the formation of a solid identity (Palen & Coatsworth, 2007). Vice-versa, the current study shed some light on the overall importance of working also on adolescents’ well-being to create the best conditions for them to face the core developmental task of building a stable identity, mainly by stimulating them toward a reflection on their emotional states, their awareness and mastery of their lives, as well as their feeling about how they are in the current multicultural society (for a review, Tejada-Gallardo et al., 2020). Moreover, working on psychological well-being has been identified as a crucial relapse-preventive strategy toward affective and anxiety disorders (Fava & Ruini, 2003), which can be a positive collateral effect of working to foster the positive loop between identity and well-being. Furthermore, stressing the remarkable role that the interplay between identity and well-being has for individuals with a migrant background, suggests that specific interventions focused on the integration of minorities are needed. Since a solid educational identity represents a crucial asset to fostering a good functioning for adolescents with a migrant background, working on integration and social support in the school context can be of outstanding importance to create more inclusive societies (Bohman & Miklikowska, 2020; Juang et al., 2021; Umaña-Taylor et al., 2018).

### Limitations and Suggestions for Future Research

The present study should be considered in light of some limitations that can suggest directions for future research. First, identity was assessed by means of questionnaires. Considering the multifaceted nature of identity, future studies should include other methodological approaches, including qualitative measures (e.g., McAdams & McLean, 2013), and assessment in different time scales (e.g., identity daily fluctuations; Klimstra & Schwab, 2021) to unfold how the interplay of identity and well-being disclose when accounting for narrative contents and short-term changes. Regarding well-being, even though it is essential to consider the perception of one’s physical health as a crucial dimension of well-being, it would be interesting to compare current results by assessing physical health with objective instruments (e.g., sleep quality through actigraphy; Natale et al., 2009) to check if it overlaps with subjective measures. It should be noted that some of the data reported in the current study have been collected during the COVID-19 pandemic, which might have played a role in adolescents’ perception of their physical health and well-being (e.g., Mancini & Imperato, 2022). Furthermore, identity formation can be addressed by using different approaches, such as a variable-centered and a person-centered one (Von Eye & Bogat, 2006), which can both be informative and enriching on this topic. The present study was conducted adopting a variable-centered approach since its purpose was to address the interplay between specific identity processes and different dimensions of well-being in adolescence. The next steps could benefit from the integration of a person-centered approach to examine the associations between well-being and different identity statuses, as well as with identity configuration emerging from the analyses of multiple domains (Crocetti et al., 2012; Luyckx et al., 2014a, 2014b). Finally, future research could benefit from disentangling the links between identity and well-being at both a between- and within-person level, by using a random-intercept cross-lagged panel model which allows for the distinction of the variance at these two different levels (Hamaker et al., 2015). Looking at the associations at multiple levels can help further understand the complex and rich interplay of identity and well-being in adolescence.

## Conclusion

Adolescents’ well-being is strongly related to the solidity of their identity and, at the same time, it can be one of the prerequisites to accomplish this crucial developmental task. Considering both identity and well-being as multifaceted constructs, a comprehensive picture of the interplay of identity processes in different domains and multiple dimensions of well-being is missing. The current longitudinal study tackled these associations, considering two different identity domains (i.e., educational and interpersonal), and multiple dimensions of positive well-being (i.e., physical health perception, subjective, psychological, and social well-being) in adolescence. A rich pattern of associations between identity processes and well-being has emerged, confirming the crucial role of identity commitment as a fundamental asset for well-being in all its facets, and highlighting the importance of considering identity from a domain-specific standpoint. This study tackled well-being as a multifaceted construct to check potential different associations between identity processes and specific dimensions of adolescents’ psychosocial adjustment. In doing so, it took a step further by accounting also for physical health perception, considering that the physical changes that individuals go across during adolescence might be determinant in the perception of their general well-being. Additionally, this study adopted a culturally sensitive approach considering both youth from the majority group and those with a migrant background, highlighting that building a solid identity, namely in the educational domain, can be a protective factor, especially for this latter group. Overall, these findings might inform future interventions to foster adolescents’ health and well-being, to support youth in the development of a solid identity, and to boost social integration in current diverse societies.

### Supplementary information

Supplementary Information
