# Supplementary material for: Identity Matters for Well-Being: The Longitudinal Associations Between Identity Processes and Well-Being in Adolescents with Different Cultural Backgrounds
Source: J Youth Adolesc. 2023 Nov 8;53(4):910–26. doi: 10.1007/s10964-023-01901-8 (PMC10879306; doi:10.1007/s10964-023-01901-8)
Supplement: Supplementary file 1 — Supplementary Information [file 10964_2023_1901_MOESM1_ESM.docx]

**Supplemental Materials for**

**Identity Matters for Well-Being: The Longitudinal Associations Between Identity Processes and Well-Being in Adolescents with Different Cultural Backgrounds**

**S1.** Sample attrition analyses……………………………………………………………………p.2

**Table S1a.** Sample attrition analyses…………………………………………………………..p. 3

**Table S1b.** Sample attrition analyses………………………………………………………...…p.4

**Table S2a.** Means, standard deviations, and correlations among study variables…………..…p. 5

**Table S2b.** Means, standard deviations, and correlations among study variables…………….p. 8

**Table S3.** Longitudinal measurement invariance…………………………………………….p. 10

**Table S4.** Cross-lagged panel models: Model fit indices and model comparison…………….p. 11

**Table S5a.** Standardized results of cross-lagged panel models………………………………..p.12

**Table S5b.** Standardized results of cross-lagged panel models: Paths with covariates….……p.17

**Table S5c.** Standardized results of cross-lagged panel models: Correlations and correlated changes………………………………………………………………………………………..p. 18

**Table S6.** Sensitivity analyses ……………………………………………...………….…….p. 21

**S1. Sample attrition analyses**

A total of 1,396 adolescents agreed to participate in the IDENTITIES project. The number of participants at each wave was as following: 1,156 at T1, 1,063 at T2, 928 at T3, and 860 at T4. To gather a better understanding of the sample attrition, additional analyses were completed to ensure that the attrition was not related to specific variables by confronting adolescents who participated in all four assessments with those who attended only three, two, or one assessment.

First, the effect of the demographic variables was checked. Boys and girls were similarly represented in the four groups. Conversely, adolescents with a migrant background were over represented compared with the expected value in the groups of adolescents who participated in 1 or 2 assessments, whereas they were underrepresented in the group of participants in all four assessments. Detailed results are available in Table S1a and S1b. Additionally, adolescents who participated in all four assessments were significantly younger than those who participated only in three, two or one assessments (*F*=5.960, *p*<.001, η^2^= .013) although the effect size of this difference was very small.

Regarding study variables, adolescents who participated in all four waves presented higher mean levels of subjective and social well-being, compared with those who only attended fewer assessments. Additionally, those who participated in all assessments showed higher commitment and in-depth exploration, and lower reconsideration of commitment in the educational domain, compared with those who attended fewer data collection. Nevertheless, the effect sizes of these differences were really small (with the percentage of explained variance ranging from 1 to 3%), thus it was possible to conclude that the different groups were rather comparable.

**Table S1a.** Sample attrition analyses for sex and background

|  | Participated in… | | | | |  |  |  |  |
| --- | --- | --- | --- | --- | --- | --- | --- | --- | --- |
|  | 4 waves  (*n* = 620) | 3 waves  (*n* = 253) | | 2 waves  (*n* = 245) | 1 wave  (*n* =278) | χ^2^ | df | *p* | Cramers’V |
| Sex |  |  |  | |  | 5.069 | 3 | .167 | .060 |
| Boys | 298 (48.1%) | 121  (47.8%) | 137  (55.9%) | | 142  (51.4%) |  |  |  |  |
| Girls | 322 (51.9%) | 132  (52.2%) | 108  (44.1%) | | 134  (48.6%) |  |  |  |  |
| Background |  |  |  | |  | **32.557** | **3** | **<.001** | **.154** |
| Italian | 528 (85.2%) | 199  (79.3%) | 175  (71.4%) | | 178  (70.9%) |  |  |  |  |
| Migrant | **92 (-)**  (14.8%) | 52  (20.7%) | **70 (+)**  (28.6%) | | **73 (+)**  (29.1%) |  |  |  |  |

*Notes:* Observed values indicated in bold are significantly different from expected values: (+) indicates that the observed value is higher than the expected value; (-) indicates that the observed value is lower than the expected value

**Table S1b.** Sample attrition analyses

|  | Participated in… | | | |  |  |  |
| --- | --- | --- | --- | --- | --- | --- | --- |
|  | 4 waves  (*n* = 620) | 3 waves  (*n* = 253) | 2 waves  (*n* = 245) | 1 wave  (*n* =278) | *F* | *p* | η^2^ |
|  | *M* (SD) | *M* (SD) | *M* (SD) | *M* (SD) |  |  |  |
| Age | 15.58_a_ (1.16) | 15.83_ab_ (1.16) | 15.91_ab_ (1.34) | 15.83_b_ (1.25) | **5.960** | **<.001** | **.013** |
| Educational commitment | 3.20_c_ (0.70) | 3.03_bc_ (0.74) | 3.09_b_ (0.78) | 2.84_a_ (0.88) | **13.209** | **<.001** | **.029** |
| Educational in-depth exploration | 3.17_c_ (0.70) | 3.09_b_ (0.74) | 3.06_bc_ (0.78) | 2.99_a_ (0.88) | **5.848** | **<.001** | **.013** |
| Educational reconsideration of commitment | 2.90_a_ (0.72) | 3.05_ab_ (0.72) | 3.06_ab_ (0.79) | 3.21_b_ (0.92) | **9.924** | **<.001** | **.022** |
| Interpersonal commitment | 3.70 (0.65) | 3.66 (0.67) | 3.60 (0.72) | 3.59 (0.84) | 1.677 | .170 | .004 |
| Interpersonal in-depth exploration | 3.46 (0.58) | 3.44 (0.62) | 3.49 (0.66) | 3.54 (0.75) | 1.104 | .346 | .003 |
| Interpersonal reconsideration of commitment | 1.97 (0.71) | 2.07 (0.77) | 2.10 (0.87) | 2.03 (1.02) | 1.696 | .166 | .004 |
| Physical health perception | 3.73 (0.60) | 3.71 (0.58) | 3.69 (0.65) | 3.63 (0.74) | 1.601 | .187 | .004 |
| Subjective well-being | 4.13_b_ (0.88) | 4.01_ab_ (1.03) | 3.99_ab_ (1.07) | 3.86_a_ (1.28) | **4.094** | **.007** | **.009** |
| Psychological well-being | 3.99 (0.89) | 3.86 (1.00) | 3.88 (1.05) | 3.83 (1,23) | 1.986 | .114 | .004 |
| Social well-being | 3.19_b_ (0.93) | 3.04_b_ (1.03) | 3.06_b_ (1.08) | 2.75_a_ (1.15) | **10.599** | **<.001** | **.023** |

*Notes.* Means were aggregated across the four waves. Means with different subscripts differ significantly (*p* < .05) as indicated by Tukey’s HSD post hoc tests

**Table S2a.** Means, standard deviations, and correlations among study variables.

|  | | *M* | *SD* | 1. | 2. | 3. | 4. | 5. | 6. | 7. | 8. | 9. | 10. | 11. | 12. | 13. | 14. | 15. | 16. | 17. | 18. | 19. | 20. | 21. | 22. |
| --- | --- | --- | --- | --- | --- | --- | --- | --- | --- | --- | --- | --- | --- | --- | --- | --- | --- | --- | --- | --- | --- | --- | --- | --- | --- |
| 1. Sex | |  |  | 1 |  |  |  |  |  |  | . |  |  |  |  |  |  |  |  |  |  |  |  |  |  |
| 2. Age | |  |  | -.00 | 1 |  |  |  |  |  |  |  |  |  |  |  |  |  |  |  |  |  |  |  |  |
| 3. COM  EDU T1 | | 3.14 | 0.83 | .01 | -.12^***^ | 1 |  |  |  |  |  |  |  |  |  |  |  |  |  |  |  |  |  |  |  |
| 4.  EXP  EDU T1 | | 3.12 | 0.68 | .11^**^ | -.02 | .58^***^ | 1 |  |  |  |  |  |  |  |  |  |  |  |  |  |  |  |  |  |  |
| 5. REC  EDU T1 | 2.96 | | 0.88 | -05 | -.13^***^ | -.30^***^ | -.02 | 1 |  |  |  |  |  |  |  |  |  |  |  |  |  |  |  |  |  |
| 6.COM INT T1 | | 3.72 | 0.77 | .06^*^ | -.09^**^ | .17^***^ | .08^*^ | -.06^*^ | 1 |  |  |  |  |  |  |  |  |  |  |  |  |  |  |  |  |
| 7.EXP INT T1 | | 3.50 | 0.71 | -22^***^ | -.05 | .04 | .17^***^ | .07^*^ | .51^***^ | 1 |  |  |  |  |  |  |  |  |  |  |  |  |  |  |  |
| 8. REC INT T1 | | 1.87 | 0.88 | -.07^*^ | -.01 | -.03 | .08^**^ | .12^***^ | -.30^***^ | -.01 | 1 |  |  |  |  |  |  |  |  |  |  |  |  |  |  |
| 9. PHWB T1 | | 3.81 | 0.65 | -.21^***^ | -.09^**^ | .16^***^ | .02 | -.05 | .14^***^ | -.06 | -.07^*^ | 1 |  |  | . |  |  |  |  |  |  |  |  |  |  |
| 10. SUBWB T1 | | 3.91 | 1.14 | -.28^***^ | -.09^**^ | .29^***^ | .08^*^ | -.13^***^ | .19^***^ | -.03 | -.09^**^ | .38^***^ | 1 |  |  |  |  |  |  |  |  |  |  |  |  |
| 11. PSYWB T1 | | 3.83 | 1.12 | -.25^***^ | -.04 | .31^***^ | .12^***^ | -.10^***^ | .31^***^ | .06^*^ | -.10^***^ | .32^***^ | .70^***^ | 1 |  |  |  |  |  |  |  |  |  |  |  |
| 12. SOCWB T1 | | 2.81 | 1.06 | -.27^***^ | -.10^***^ | .27^***^ | .11^***^ | -.10^**^ | .29^***^ | .06^*^ | -.02 | .28^***^ | .60^***^ | .65^***^ | 1 |  |  |  |  |  |  |  |  |  |  |
| 13. COM EDU T2 | | 3.16 | 0.82 | -.01 | -11^***^ | .64^***^ | .38^***^ | -.23^***^ | .14^***^ | .03 | .04 | .12^***^ | .22^***^ | .25^***^ | .22^***^ | 1 |  |  |  |  |  |  |  |  |  |
| 14.EXP EDU T2 | | 3.52 | 0.70 | .14^***^ | .05 | .39^***^ | .58^***^ | -.05 | .02 | .16^***^ | .09^**^ | -.02 | .06 | .10^**^ | .09^**^ | .52^***^ | 1 |  |  |  |  |  |  |  |  |
| 15. REC EDU T2 | | 3.03 | 0.88 | -.07^*^ | .07^*^ | -.21^***^ | -.07^*^ | .57^***^ | -.02 | .10^**^ | .15^***^ | -.09^**^ | -.08^*^ | -.04 | -.07^*^ | -.24^***^ | -.02 | 1 |  |  |  |  |  |  |  |
| 16. COM INT T2 | | 3.69 | 0.80 | -02 | .01 | .18^***^ | -.08^*^ | -04 | .57^***^ | .33^***^ | -.21^***^ | .08^*^ | .20^***^ | .27^***^ | .26^***^ | .22^***^ | .13^***^ | .06^*^ | 1 |  |  |  |  |  |  |
| 17. EXP INT T2 | | 3.52 | 0.72 | .22^***^ | .02 | .05 | .15^***^ | .09^**^ | .35^***^ | .58^***^ | -.05 | -.07^*^ | -.07^*^ | .05 | .02 | .09^**^ | .25^***^ | .18^***^ | .51^***^ | 1 |  |  |  |  |  |
| 18. REC INT T2 | | 2.05 | 0.95 | -.04 | -.02 | -.07^*^ | .02 | .11^**^ | -.13^***^ | .02 | .47^***^ | -.06 | -.09^*^ | -.03 | .01 | .05 | .09^**^ | .18^***^ | -.19^***^ | .09^**^ | 1 |  |  |  |  |
| 19. PHWB T2 | | 3.73 | 0.71 | -.17^***^ | -.15^***^ | .17^***^ | .01 | -.05 | .15^***^ | -.06 | -.05 | .63^***^ | .33^***^ | .29^***^ | .25^***^ | .19^***^ | .04 | .17^*^ | .13^***^ | -.05 | -.11^***^ | 1 |  |  |  |
| 20. SUBWB T2 | | 4.06 | 1.13 | -.25^***^ | -.08^*^ | .26^***^ | .07^*^ | -.07^*^ | .21^***^ | -.01 | -.08^*^ | .29^***^ | .61^***^ | .57^***^ | .48^***^ | .34^***^ | .10^***^ | -.07^*^ | .27^***^ | .00 | -.02 | .32^***^ | 1 |  |  |
| 21. PSYWB T2 | | 3.91 | 1.14 | -.24^***^ | -.05 | .33^***^ | .15^***^ | -.06 | .27^***^ | .03 | -.08* | .27^***^ | .54^***^ | .64^***^ | .50^***^ | .36^***^ | .20^***^ | -.03 | .34^***^ | .08^*^ | -.03 | .31^***^ | .76^***^ | 1 |  |
| 22. SOCWB T2 | | 3.13 | 1.21 | -.24^***^ | -.09^**^ | .30^***^ | .15^***^ | -.04 | .23^***^ | .05 | -.03 | .22^***^ | .45^***^ | .51^***^ | .57^***^ | .36^***^ | .19^***^ | -.00 | .31^***^ | .10^**^ | .07^*^ | .22^***^ | .66^***^ | .71^***^ | 1 |
| 23. COM EDU T3 | | 3.14 | 0.84 | -.01 | -.05 | .60^***^ | .37^***^ | -.28^***^ | .09^*^ | .01 | .04 | .11^**^ | .21^***^ | .22^***^ | .25^***^ | .68^***^ | 44^***^ | -.27^***^ | .19^***^ | .08^*^ | -.05 | .15^***^ | .27^***^ | .33^***^ | .37^***^ |
| 24. EXP EDU T3 | | 3.15 | 0.72 | .11^**^ | .01 | .39^***^ | .51^***^ | -.11^**^ | .10^**^ | .12^***^ | .12^***^ | .00 | .07 | .09^**^ | .15^***^ | .40^***^ | .59^***^ | -.16^***^ | .10^**^ | .16^***^ | .08^*^ | .04 | .06 | .18^***^ | .20^***^ |
| 25. REC EDU T3 | | 2.95 | 0.89 | -.10^**^ | .07^*^ | -.26^***^ | -.12^***^ | .50^***^ | .05 | .04 | .11^**^ | -.07^*^ | -.06 | -.04 | -.00 | -.29^***^ | -.12^***^ | .59^***^ | .04 | .10^**^ | .14^***^ | -.06 | -.08^*^ | -.03 | -.01 |
| 26. COM INT T3 | | 3.67 | 0.80 | -.04 | .02 | .14^***^ | .06 | -.03 | .51^***^ | .30^***^ | .18^***^ | .08^*^ | .19^***^ | .23^***^ | .24^***^ | .17^***^ | .09^*^ | .02 | .61^***^ | .33^***^ | -.13^***^ | .11^**^ | .25^***^ | .34^***^ | .28^***^ |
| 27. EXP INT T3 | | 3.48 | 0.73 | -15^***^ | .05 | .05 | .13^***^ | .07 | .36^***^ | .49^***^ | -.06 | -.05 | .01 | .08^*^ | .09^*^ | .06 | .20^***^ | .12^***^ | .39^***^ | .57^***^ | -.02 | -.04 | .06 | .16^***^ | .15^***^ |
| 28. REC INT T3 | | 2.05 | 0.97 | -.10^**^ | -.01 | -.07 | -.03 | .13^***^ | -.18^***^ | -.01 | .37^***^ | -.02 | -.05 | -.07^*^ | -.09^*^ | -.01 | .03 | .18^***^ | -.16^***^ | .01 | .46^***^ | -.10^**^ | -.07 | -.05 | .02 |
| 29. PHWB T3 | | 3.65 | 0.75 | -.21^***^ | -.13^***^ | .08^*^ | -.04 | -.03 | .12^***^ | -.04 | .-.06 | .59^***^ | .30^***^ | .27^***^ | .23^***^ | .13^***^ | .01 | -.01 | .14^***^ | -.01 | -.06 | .66^***^ | .27^***^ | .27^***^ | .20^***^ |
| 30. SUBWB T3 | | 4.19 | 1.14 | -.19^***^ | -.05 | .25^***^ | .08^*^ | -.05 | .18^***^ | .02 | -.00 | .21^***^ | .55^***^ | .50^***^ | .44^***^ | .26^***^ | .11^**^ | -.01 | .27^***^ | .04 | -.04 | .29^***^ | .63^***^ | .57^***^ | .49^***^ |
| 31. PSYWB T3 | | 4.03 | 1.13 | -.21^***^ | -.03 | .24^***^ | .10^**^ | -.04 | .19^***^ | .03 | -.05 | .24^***^ | .48^***^ | .54^***^ | .45^***^ | .25^***^ | .11^**^ | -.04 | .27^***^ | .05 | -.06 | .26^***^ | .55^***^ | .61^***^ | .49^***^ |
| 32. SOCWB T3 | | 3.25 | 1.26 | -.25^***^ | -.09^**^ | .21^***^ | .12^***^ | .02 | .19^***^ | .06 | .02 | .23^***^ | .41^***^ | .44^***^ | .49^***^ | .24^***^ | .14^***^ | .03 | .28^***^ | .10^**^ | .03 | .22^***^ | .49^***^ | .50^***^ | .60^***^ |
| 33. COM EDU T4 | | 3.10 | 0.86 | .01 | -.13^***^ | .53^***^ | .32^***^ | -.28^***^ | .15^***^ | .05 | -.04 | .11^**^ | .17^***^ | .20^***^ | .22^***^ | .60^***^ | .33^***^ | -.30^***^ | .19^***^ | .10^*^ | -.01 | .17^***^ | .24^***^ | .34^***^ | .31^***^ |
| 34. EXP EDU T4 | | 3.11 | 0.69 | .10^**^ | -.04 | .35^***^ | .46^***^ | -.09^*^ | .16^***^ | .18^***^ | -.04 | .03 | .11^**^ | .13^***^ | .18^***^ | .36^***^ | .54^***^ | -.09^*^ | .18^***^ | .17^***^ | .08^*^ | .09^*^ | .11^**^ | .21^***^ | .19^***^ |
| 35. REC EDU T4 | | 2.89 | 0.92 | -.09^**^ | .08^*^ | -.24^***^ | -.12^**^ | .47^***^ | .05 | .07 | .10^*^ | -.07 | -.06 | -.02 | -.04 | -.28^***^ | -.10^**^ | .56^***^ | .03 | .08^*^ | .13^***^ | -.06 | -.06 | -.02 | .00 |
| 36. COM INT T4 | | 3.60 | 0.83 | .00 | -.02 | .14^***^ | .06 | -.06 | .51^***^ | .32^***^ | -.20^***^ | .14^***^ | .19^***^ | .26^***^ | .29^***^ | .18^***^ | .06 | -.03 | .52^***^ | .34^***^ | -.19^***^ | .16^***^ | .24^***^ | .32^***^ | .30^***^ |
| 37. EXP INT T4 | | 3.34 | 0.74 | .11^**^ | -.03 | .10^*^ | .15^***^ | -.01 | .32^***^ | .48^***^ | -.04 | .02 | .04 | .09^*^ | .12^**^ | .11^**^ | .21^***^ | .02 | .30^***^ | .46^***^ | .02 | -.03 | .05 | .13^***^ | .10^**^ |
| 38. REC INT T4 | | 2.06 | 1.00 | -.06 | -.05 | -.10^*^ | -.04 | .10^**^ | -.12^**^ | -.01 | .34^***^ | -.08^*^ | -.08^*^ | -.12^**^ | -.08^*^ | -.03 | .05 | .16^***^ | -.12^**^ | -.01 | .43^***^ | -.11^**^ | -.10^*^ | -.08^*^ | -.00 |
| 39. PHWB T4 | | 3.64 | 0.74 | -.19^***^ | -.14^***^ | .10^**^ | -.04 | -.03 | .08^*^ | -.10^**^ | -.05 | .57^***^ | .27^***^ | .26^***^ | .22^***^ | .18^***^ | .00 | -.06 | .07 | -.09^*^ | -.08^*^ | .64^***^ | .28^***^ | .32^***^ | .21^***^ |
| 40. SUBWB T4 | | 4.19 | 1.03 | -.22^***^ | -.04 | .19^***^ | .02 | -.08^*^ | .20^***^ | -.01 | -.05 | .25^***^ | .52^***^ | .49^***^ | .40^***^ | .31^***^ | .09^*^ | -.05 | .20^***^ | .01 | -.01 | .28^***^ | .52^***^ | .50^***^ | .42^***^ |
| 41. PSYWB T4 | | 4.05 | 1.05 | -.18^***^ | -.02 | .18^***^ | .03 | -.08^*^ | .24^***^ | .03 | -.05 | .22^***^ | .41^***^ | .49^***^ | .41^***^ | .32^***^ | .12^**^ | -.10^**^ | .26^***^ | .07^*^ | -.02 | .31^***^ | .48^***^ | .58^***^ | .44^***^ |
| 42. SOCWB T4 | | 3.41 | 1.12 | -.24^***^ | -.07 | .21^***^ | .01 | -.09^*^ | .23^***^ | .00 | .00 | .22^***^ | .39^***^ | .44^***^ | .50^***^ | .35^***^ | .16^***^ | -.07 | .26^***^ | .06 | .05 | .24^***^ | .46^***^ | .53^***^ | .58^***^ |

*Note*. T = Time. Sex: 0 = Boys, 1 = Girls. COM EDU = educational commitment; EXP EDU= educational in-depth exploration; REC EDU= educational reconsideration of commitment; COM INT= interpersonal commitment; EXP INT= interpersonal in-depth exploration; REC INT= interpersonal reconsideration of commitment; PHWB= physical health perception; SUBWB= subjective well-being; PSYWB= psychological well-being; SOCWB= social well-being.

^*^ *p* < .05; ^*^ *p* < .01^*^; ^***^ *p* < .001.

**Table S2b.** Means, standard deviations, and correlations among study variables.

|  | 23. | 24. | 25. | 26. | 27. | 28. | 29. | 30. | 31. | 32. | 33. | 34. | 35. | 36. | 37. | 38. | 39. | 40. | 41. | 42. |  |
| --- | --- | --- | --- | --- | --- | --- | --- | --- | --- | --- | --- | --- | --- | --- | --- | --- | --- | --- | --- | --- | --- |
| 43. COM EDU T3 | 1 |  |  |  |  |  |  |  |  |  |  |  |  |  |  |  |  |  |  |  | |
| 44. EXP EDU T3 | .62^***^ | 1 |  |  |  |  |  |  |  |  |  |  |  |  |  |  |  |  |  |  | |
| 45. REC EDU T3 | -.26^***^ | -.05 | 1 |  |  |  |  |  |  |  |  |  |  |  |  |  |  |  |  |  | |
| 46. COM INT T3 | .23^***^ | .16^***^ | .05 | 1 |  |  |  |  |  |  |  |  |  |  |  |  |  |  |  |  | |
| 47. EXP INT T3 | .12^***^ | .26^***^ | .14^***^ | .54^***^ | 1 |  |  |  |  |  |  |  |  |  |  |  |  |  |  |  | |
| 48. REC INT T3 | -.05 | .07^*^ | .14^***^ | -22^***^ | .07^*^ | 1 |  |  |  |  |  |  |  |  |  |  |  |  |  |  | |
| 49. PHWB T3 | .15^***^ | .02 | -.05 | .13^***^ | -.03 | -.12^***^ | 1 |  |  |  |  |  |  |  |  |  |  |  |  |  | |
| 50. SUBWB T3 | .34^***^ | .17^***^ | -.08^*^ | .27^***^ | .05 | -.09^*^ | .30^***^ | 1 |  |  |  |  |  |  |  |  |  |  |  |  | |
| 51. PSYWB T3 | .34^***^ | .20^***^ | -.04 | .30^***^ | .12^**^ | -.12^***^ | .32^***^ | .75^***^ | 1 |  |  |  |  |  |  |  |  |  |  |  | |
| 52. SOCWB T3 | .32^***^ | .17^***^ | .01 | .31^***^ | .15^***^ | .02 | .24^***^ | .66^***^ | .72^***^ | 1 |  |  |  |  |  |  |  |  |  |  | |
| 53. COM EDU T4 | .72^***^ | .47^***^ | -.33^***^ | .20^***^ | .12^***^ | -.02 | .12^**^ | .29^***^ | .30^***^ | .30^***^ | 1 |  |  |  |  |  |  |  |  |  | |
| 54. EXP EDU T4 | .48^***^ | .60^***^ | -.12^**^ | .18^***^ | .20^***^ | .05 | .05 | .21^***^ | .22^***^ | .22^***^ | .61^***^ | 1 |  |  |  |  |  |  |  |  | |
| 55. REC EDU T4 | -.26^***^ | -.06 | .62^***^ | .02 | .15^***^ | .14^***^ | -.03 | -.04 | -.02 | -.00 | -.30^***^ | -.00 | 1 |  |  |  |  |  |  |  | |
| 56. COM INT T4 | .21^***^ | .14^***^ | .03 | .62^***^ | .37^***^ | -.27^***^ | .14^***^ | .25^***^ | .27^***^ | .30^***^ | .23^***^ | .19^***^ | .02 | 1 |  |  |  |  |  |  | |
| 57. EXP INT T4 | .13^***^ | .23^***^ | .06 | .35^***^ | .58^***^ | -.02 | -.03 | .07 | .12^***^ | .16^***^ | .18^***^ | .30^***^ | .08^*^ | .52^***^ | 1 |  |  |  |  |  | |
| 58. REC INT T4 | .00 | .07^*^ | .13^***^ | -.20^***^ | -.02 | .53^***^ | -.12^**^ | -.07 | -.11^**^ | -.01 | .01 | .11^**^ | .25^***^ | -.25^***^ | .09^**^ | 1 |  |  |  |  | |
| 59. PHWB T4 | .10^**^ | -.02 | -.05 | .09^*^ | -.08^*^ | -.09^*^ | .69^***^ | .28^***^ | .28^**^ | .19^***^ | .16^***^ | .04 | -.07^*^ | .18^***^ | -.02 | -.11^**^ | 1 |  |  |  | |
| 60. SUBWB T4 | .29^***^ | .14^***^ | -.11^**^ | .23^***^ | .02 | -.05 | .29^***^ | .61^***^ | .53^**^ | .44^***^ | .32^***^ | .17^***^ | .09^**^ | .28^***^ | .10^**^ | -.09^*^ | .34^***^ | 1 |  |  | |
| 61. PSYWB T4 | .33^***^ | .17^***^ | -11^**^ | .26^***^ | .07 | -.07 | .30^***^ | .59^***^ | .60^**^ | .49^***^ | .36^***^ | .19^***^ | -.07 | .32^***^ | .13^***^ | -.08^*^ | .32^***^ | .73^***^ | 1 |  | |
| 62. SOCWB T4 | .36^***^ | .20^***^ | -.05 | .28^***^ | .12^**^ | -.01 | .24^***^ | .50^***^ | .52^**^ | .60^***^ | .39^***^ | .25^***^ | -.05 | .32^***^ | .12^***^ | .01 | .25^***^ | .65^***^ | .70^***^ | 1 | |

*Note*. T = Time. Sex: 0 = Boys, 1 = Girls. COM EDU = educational commitment; EXP EDU= educational in-depth exploration; REC EDU= educational reconsideration of commitment; COM INT= interpersonal commitment; EXP INT= interpersonal in-depth exploration; REC INT= interpersonal reconsideration of commitment; PHWB= physical health perception; SUBWB= subjective well-being; PSYWB= psychological well-being; SOCWB= social well-being.

^*^ *p* <.05; ^**^ *p* < .01; ^***^ *p* < .001.

**Table S3.** Longitudinal measurement invariance

| Educational identity processes | Model fit | | | | | |  | Model comparisons | | | |
| --- | --- | --- | --- | --- | --- | --- | --- | --- | --- | --- | --- |
|  | χ_SB_^2^ | df | CFI | TLI | SRMR | RMSEA [90% CI] |  | Models | Δχ_SB_^2^(Δdf) | ΔCFI | ΔRMSEA |
| Configural invariance (M1) | 2038.873 | 1130 | .932 | .920 | .053 | .033 [.031, .036] |  |  |  |  |  |
| Metric invariance (M2) | 2078.056 | 1160 | .931 | .921 | .054 | .033 [.031, .035] |  | M2-M1 | 34.944 (30) | -.001 | .000 |
| Interpersonal identity processes |  |  |  |  |  |  |  |  |  |  |  |
| Configural invariance (M1) | 2124.815 | 1130 | .933 | .921 | .056 | .035 [.033, .037] |  |  |  |  |  |
| Metric invariance (M2) | 2156.226 | 1160 | .932 | .922 | .058 | .035 [.033, .037] |  | M2-M1 | 37.274 (30) | -.001 | .000 |
| Physical health perception |  |  |  |  |  |  |  |  |  |  |  |
| Configural invariance (M1) | 144.617 | 74 | .974 | .959 | .033 | .036 [.027, .045] |  |  |  |  |  |
| Metric invariance (M2) | 151.961 | 83 | .975 | .964 | .038 | .034 [.025, .042] |  | M2-M1 | 5.921 (9) | .001 | -.002 |
| Subjective, psychological, and social well-being |  |  |  |  |  |  |  |  |  |  |  |
| Configural invariance (M1) | 2500.635 | 1334 | .921 | .908 | .059 | .035 [.033, .037] |  |  |  |  |  |
| Metric invariance (M2) | 2593.649 | 1367 | .916 | .906 | .063 | .035 [.033, .037] |  | M2-M1 | 81.450 (33) | -.005 | .000 |

*Note*. χ_SB_^2^ = Satorra-Bentler scaled chi-square; df = degree of freedom; CFI = Comparative Fit Index; TLI = Tucker-Lewis Index; SRMR = Standardized Root Mean Square Residual; RMSEA = Root Mean Square Error of Approximation; CI = confidence interval; Δ = change in the parameter.

^*^ *p* < 0.05; ^**^ *p* < 0.01; ^***^ *p* < 0.001

**Table S4.** Cross-lagged panel models: Model fit indices and model comparison

| Model 1- Identity and physical health perception | Model fit | | | | | |  | Model comparisons | | | |
| --- | --- | --- | --- | --- | --- | --- | --- | --- | --- | --- | --- |
|  | χ_SB_^2^ | df | CFI | TLI | SRMR | RMSEA [90% CI] |  | Models | Δχ_SB_^2^ | ΔCFI | ΔRMSEA |
| Unconstrained (M1) | 382.374 | 147 | .956 | .869 | .039 | .048 [.042, .053] |  |  |  |  |  |
| Cross-lagged paths fixed (M2) | 488.318 | 231 | .952 | .909 | .045 | .040 [.035, .045] |  | M2-M1 | 105.627 (84) | -.004 | -.008 |
| Cross-lagged paths and within time correlations fixed (M3) | 528.015 | 273 | .952 | .924 | .047 | .036 [.032, .041] |  | M3-M2 | 44.593 (42) | .000 | -.008 |
| Model 2 - Identity and subjective well-being |  |  |  |  |  |  |  |  |  |  |  |
| Unconstrained (M1) | 367.075 | 147 | .958 | .877 | .039 | .046 [.040, .052] |  |  |  |  |  |
| Cross-lagged paths fixed (M2) | 462.146 | 231 | .956 | .918 | .045 | .038 [.033, .043] |  | M2-M1 | 95.683 (84) | -.002 | -.008 |
| Cross-lagged paths and within time correlations fixed (M3) | 491.071 | 273 | .959 | .934 | .046 | .034 [.029, .038] |  | M3-M2 | 34.775 (42) | .003 | -.004 |
| Model 3 - Identity and psychological well-being |  |  |  |  |  |  |  |  |  |  |  |
| Unconstrained (M1) | 369.726 | 147 | .958 | .875 | .038 | .046 [.040, .052] |  |  |  |  |  |
| Cross-lagged paths fixed (M2) | 471.627 | 231 | .954 | .914 | .045 | .038 [.033, .043] |  | M2-M1 | 102.741 (84) | -.002 | -.008 |
| Cross-lagged paths and within time correlations fixed (M3) | 500.707 | 273 | .957 | .931 | .046 | .034 [.030, .039] |  | M3-M2 | 35.528 (42) | .003 | -.004 |
| Model 4 - Identity and social well-being |  |  |  |  |  |  |  |  |  |  |  |
| Unconstrained (M1) | 386.877 | 147 | .955 | .867 | .040 | .048 [.042, .054] |  |  |  |  |  |
| Cross-lagged paths fixed (M2) | 487.478 | 231 | .952 | .910 | .046 | .040 [.035, .045] |  | M2-M1 | 102.136 (84) | -.003 | -.008 |
| Cross-lagged paths and within time correlations fixed (M3) | 515.495 | 273 | .954 | .928 | .048 | .035 [.031,.040] |  | M3-M2 | 39.326 (42) | .002 | -.005 |

*Note*. χ_SB_^2^ = Satorra-Bentler scaled chi-square; df = degree of freedom; CFI = Comparative Fit Index; TLI = Tucker-Lewis Index; SRMR = Standardized Root Mean Square Residual; RMSEA = Root Mean Square Error of Approximation; CI = confidence interval; Δ = change in the parameter.

^*^ *p* < 0.05; ^**^ *p* < 0.01; ^***^ *p* < 0.001

**Table S5a.** Standardized results of the cross-lagged panel models: Cross-lagged paths

|  | Model 1 - Identity and physical health perception | | | Model 2 - Identity and subjective well-being | | | Model 3 - Identity and psychological well-being | | | Model 4 - Identity and social well-being | | |
| --- | --- | --- | --- | --- | --- | --- | --- | --- | --- | --- | --- | --- |
|  | T1🡪T2 | T2🡪T3 | T3🡪T4 | T1🡪T2 | T2🡪T3 | T3🡪T4 | T1🡪T2 | T2🡪T3 | T3🡪T4 | T1🡪T2 | T2🡪T3 | T3🡪T4 |
| Educational commitment → Educational in-depth exploration | .14^***^ | .14^***^ | .14^**^ | .13^***^ | .13^***^ | .14^***^ | .12^***^ | .13^***^ | .13^***^ | .12^***^ | .13^***^ | .13^***^ |
| Educational commitment → Educational reconsideration of commitment | -.10^***^ | -.10^***^ | -.09^***^ | -.10^***^ | -.10^***^ | -.10^***^ | -.11^***^ | -.11^***^ | -.10^***^ | -.10^***^ | -.10^***^ | -.10^***^ |
| Educational commitment → Interpersonal commitment | .08^***^ | .08^***^ | .71^***^ | .06^**^ | .06^**^ | .06^**^ | .05^*^ | .05^*^ | .05^*^ | .06^*^ | .06^*^ | .05^*^ |
| Educational commitment → Interpersonal in-depth exploration | -.01 | -.01 | -.01 | -.01 | -.01 | -.01 | -.03 | -.03 | -.03 | -.02 | -.02 | -.02 |
| Educational commitment → Interpersonal reconsideration of commitment | -.01 | -.01 | -.01 | -.01 | -.00 | -.00 | -.01 | -.01 | -.01 | -.02 | -.02 | -.02 |
| Educational commitment → Physical health perception | .04 | .04 | .03 |  |  |  |  |  |  |  |  |  |
| Educational commitment → Subjective well-being |  |  |  | .08^***^ | .08^***^ | .09^***^ |  |  |  |  |  |  |
| Educational commitment → Psychological well-being |  |  |  | . |  |  | .09^***^ | .09^***^ | .10^***^ |  |  |  |
| Educational commitment → Social well-being |  |  |  |  |  |  |  |  |  | .10^***^ | .10^***^ | .11^***^ |
| Educational in-depth exploration → Educational commitment | .09^***^ | .10^***^ | .09^***^ | .09^***^ | .10^***^ | .09^***^ | .09^***^ | .09^***^ | .09^***^ | .09^***^ | .10^***^ | .09^***^ |
| Educational in-depth exploration → Educational reconsideration of commitment | -.01 | -.01 | -.01 | -.01 | -.01 | -.01 | -.01 | -.01 | -.01 | -.01 | -.01 | -.01 |
| Educational in-depth exploration → Interpersonal commitment | -.02 | -.02 | -.02 | -.02 | -.02 | -.02 | -.03 | -.03 | -.03 | -.02 | -.03 | -.02 |
| Educational in-depth exploration → Interpersonal in-depth exploration | .05^**^ | .05^**^ | .05^**^ | .05^**^ | .06^**^ | .05^**^ | .05^*^ | .05^*^ | .05^*^ | .05^*^ | .05^*^ | .05^*^ |
| Educational in-depth exploration → Interpersonal reconsideration of commitment | .02 | .02 | .02 | .02 | .02 | .02 | .02 | .02 | .02 | .02 | .02 | .02 |
| Educational in-depth exploration → Physical health perception | -.01 | -.01 | -.01 |  |  |  |  |  |  |  |  |  |
| Educational in-depth exploration → Subjective well-being |  |  |  | .01 | .01 | .01 |  |  |  |  |  |  |
| Educational in-depth exploration → Psychological well-being |  |  |  |  |  |  | .01 | .01 | .02 |  |  |  |
| Educational in-depth exploration → Social well-being |  |  |  |  |  |  |  |  |  | .03 | .03 | .03 |
| Educational reconsideration of commitment → Educational commitment | -.10^***^ | -10^***^ | -.10^***^ | -.10^***^ | -.10^***^ | -.10^***^ | -.10^***^ | -.10^***^ | -10^***^ | -.10^***^ | -10^***^ | -.10^***^ |
| Educational reconsideration of commitment → Educational in-depth exploration | -.04 | -.04 | -.04 | -.04 | -.04 | -.04 | -.04^*^ | -.04^*^ | -.04^*^ | -.04^*^ | -.04^*^ | -.04^*^ |
| Educational reconsideration of commitment → Interpersonal commitment | .01 | .01 | .01 | .01 | .01 | .01 | .01 | .01 | .01 | .01 | .01 | .01 |
| Educational reconsideration of commitment → Interpersonal in-depth exploration | .03 | .03 | .03 | .03 | .03 | .03 | .03 | .03 | .03 | .03 | .03 | .03 |
| Educational reconsideration of commitment → Interpersonal reconsideration of commitment | .06^**^ | .06^**^ | .06^**^ | .06^**^ | .06^***^ | .06^**^ | .06^**^ | .06^**^ | .06^**^ | .06^**^ | .06^**^ | .06^**^ |
| Educational reconsideration of commitment → Physical health perception | .01 | .01 | .01 |  |  |  |  |  |  |  |  |  |
| Educational reconsideration of commitment → Subjective well-being |  |  |  | -.01 | -.01 | -.01 |  |  |  |  |  |  |
| Educational reconsideration of commitment → Psychological well-being |  |  |  |  |  |  | -.01 | -.01 | -.01 |  |  |  |
| Educational reconsideration of commitment → Social well-being |  |  |  |  |  |  |  |  |  | .02 | .02 | .02 |
| Interpersonal commitment → Educational commitment | .06^**^ | .06^**^ | .06^**^ | .05^*^ | .06^**^ | .05^*^ | .04^*^ | .05^*^ | .05^*^ | .04 | .04 | .04 |
| Interpersonal commitment → Educational in-depth exploration | .02 | .02 | .02 | .01 | .01 | .01 | .00 | .00 | .00 | .00 | .00 | .00 |
| Interpersonal commitment → Educational reconsideration of commitment | .02 | .02 | .02 | .02 | .02 | .02 | .01 | .01 | .01 | .02 | .02 | .02 |
| Interpersonal commitment → Interpersonal in-depth exploration | .11^***^ | .11^**^ | .11^***^ | .11^***^ | .11^***^ | .11^***^ | .09^***^ | .09^***^ | .09^***^ | .10^***^ | .10^***^ | .10^***^ |
| Interpersonal commitment → Interpersonal reconsideration of commitment | -.03 | -.03 | -.03 | -.03 | -.03 | -.03 | -.03 | -.03 | -.03 | -.04 | -.04 | -.04 |
| Interpersonal commitment → Physical health perception | .05^**^ | .05^**^ | .05^**^ |  |  |  |  |  |  |  |  |  |
| Interpersonal commitment → Subjective well-being |  |  |  | .07^***^ | .08^***^ | .08^***^ |  |  |  |  |  |  |
| Interpersonal commitment → Psychological well-being |  |  |  |  |  |  | .08^***^ | .09^***^ | .09^***^ |  |  |  |
| Interpersonal commitment → Social well-being |  |  |  |  |  |  |  |  |  | .07^***^ | .07^***^ | .08^***^ |
| Interpersonal in-depth exploration → Educational commitment | -.03 | -.03 | -.03 | -.02 | -.02 | -.02 | -.02 | -.02 | -.02 | -.03 | -.03 | -.03 |
| Interpersonal in-depth exploration → Educational in-depth exploration | .03 | .03 | .03 | .03 | .03 | .03 | .03 | .03 | .03 | .03 | .03 | .03 |
| Interpersonal in-depth exploration → Educational reconsideration of commitment | .07^**^ | .07^**^ | .06^**^ | .07^**^ | .07^**^ | .07^**^ | .07^**^ | .07^***^ | .07^**^ | .07^**^ | .07^**^ | .07^**^ |
| Interpersonal in-depth exploration → Interpersonal commitment | .10^***^ | .10^***^ | .10^***^ | .11^***^ | .11^***^ | .10^**^ | .10^***^ | .09^***^ | .10^***^ | .10^***^ | .10^***^ | .10^***^ |
| Interpersonal in-depth exploration → Interpersonal reconsideration of commitment | .01 | .01 | .01 | .01 | .01 | .01 | .01 | .01 | .01 | .02 | .01 | .01 |
| Interpersonal in-depth exploration → Physical health perception | -.05^*^ | -.05^*^ | -.05^*^ |  |  |  |  |  |  |  |  |  |
| Interpersonal in-depth exploration → Subjective well-being |  |  |  | -.01 | -.01 | -.01 |  |  |  |  |  |  |
| Interpersonal in-depth exploration → Psychological well-being |  |  |  |  |  |  | -.02 | -.02 | -.02 |  |  |  |
| Interpersonal in-depth exploration → Social well-being |  |  |  |  |  |  |  |  |  | -.01 | -.01 | -.01 |
| Interpersonal reconsideration of commitment → Educational commitment | .03 | .03 | .03 | .03 | .03 | .03 | .03 | .03 | .03 | .02 | .02 | .02 |
| Interpersonal reconsideration of commitment → Educational in-depth exploration | .05^**^ | .06^**^ | .06^**^ | .05^**^ | .06^**^ | .06^**^ | .05^**^ | .06^**^ | .06^**^ | .05^**^ | .05^**^ | .06^**^ |
| Interpersonal reconsideration of commitment → Educational reconsideration of commitment | .06^***^ | .07^***^ | .07^***^ | .06^***^ | .07^***^ | .07^***^ | .06^***^ | .07^***^ | .07^***^ | .06^***^ | .07^***^ | .07^***^ |
| Interpersonal reconsideration of commitment → Interpersonal commitment | -.06^**^ | -.06^**^ | -.06^**^ | -.06^**^ | -.06^**^ | -.06^**^ | -.06^**^ | -.06^**^ | -.06^**^ | -.06^***^ | -.07^***^ | -.07^***^ |
| Interpersonal reconsideration of commitment → Interpersonal in-depth exploration | -.02 | -.02 | -.02 | -.02 | -.02 | -.02 | .01 | -.02 | -.02 | -.02 | -.02 | -.02 |
| Interpersonal reconsideration of commitment → Physical health perception | -.00 | -.00 | -.00 |  |  |  |  |  |  |  |  |  |
| Interpersonal reconsideration of commitment → Subjective well-being |  |  |  | .00 | .00 | .00 |  |  |  |  |  |  |
| Interpersonal reconsideration of commitment → Psychological well-being |  |  |  |  |  |  | .00 | .00 | .01 |  |  |  |
| Interpersonal reconsideration of commitment → Social well-being |  |  |  |  |  |  |  |  |  | .00 | .00 | .00 |
| Physical health perception → Educational commitment | .02 | .03 | .03 |  |  |  |  |  |  |  |  |  |
| Physical health perception → Educational in-depth exploration | .01 | .01 | .01 |  |  |  |  |  |  |  |  |  |
| Physical health perception → Educational reconsideration of commitment | -.02 | -.02 | -.02 |  |  |  |  |  |  |  |  |  |
| Physical health perception → Interpersonal commitment | .02 | .02 | .02 |  |  |  |  |  |  |  |  |  |
| Physical health perception → Interpersonal in-depth exploration | -.03^*^ | -.03^*^ | -.03^*^ |  |  |  |  |  |  |  |  |  |
| Physical health perception → Interpersonal reconsideration of commitment | -.05^**^ | -.06^**^ | -.06^**^ |  |  |  |  |  |  |  |  |  |
| Subjective well-being → Educational commitment |  |  |  | .05^**^ | .05^**^ | .05^**^ |  |  |  |  |  |  |
| Subjective well-being → Educational in-depth exploration |  |  |  | .02 | .02 | .02 |  |  |  |  |  |  |
| Subjective well-being → Educational reconsideration of commitment |  |  |  | .01 | .01 | .01 |  |  |  |  |  |  |
| Subjective well-being → Interpersonal commitment |  |  |  | .06^**^ | .06^**^ | .06^**^ |  |  |  |  |  |  |
| Subjective well-being → Interpersonal in-depth exploration |  |  |  | -.00 | -.00 | -.00 |  |  |  |  |  |  |
| Subjective well-being → Interpersonal reconsideration of commitment |  |  |  | -.04 | -.03 | -.03 |  |  |  |  |  |  |
| Psychological well-being → Educational commitment |  |  |  |  |  |  | .06^***^ | .06^***^ | .06^***^ |  |  |  |
| Psychological well-being → Educational in-depth exploration |  |  |  |  |  |  | .05^**^ | .05^**^ | .06^**^ |  |  |  |
| Psychological well-being → Educational reconsideration of commitment |  |  |  |  |  |  | .03 | .03 | .03 |  |  |  |
| Psychological well-being → Interpersonal commitment |  |  |  |  |  |  | .10^***^ | .10^***^ | .10^***^ |  |  |  |
| Psychological well-being → Interpersonal in-depth exploration |  |  |  |  |  |  | .05^*^ | .05^*^ | .05^*^ |  |  |  |
| Psychological well-being → Interpersonal reconsideration of commitment |  |  |  |  |  |  | -.02 | -.02 | -.02 |  |  |  |
| Social well-being → Educational commitment |  |  |  |  |  |  |  |  |  | .06^***^ | .07^***^ | .07^***^ |
| Social well-being → Educational in-depth exploration |  |  |  |  |  |  |  |  |  | .04^**^ | .05^**^ | .06^**^ |
| Social well-being → Educational reconsideration of commitment |  |  |  |  |  |  |  |  |  | .00 | .00 | .00 |
| Social well-being → Interpersonal commitment |  |  |  |  |  |  |  |  |  | .08^***^ | .09^***^ | .09^***^ |
| Social well-being → Interpersonal in-depth exploration |  |  |  |  |  |  |  |  |  | .02 | .03 | .03 |
| Social well-being → Interpersonal reconsideration of commitment |  |  |  |  |  |  |  |  |  | .01 | .01 | .01 |

*Notes*: T=Time

^*^ *p* < 0.05; ^**^ *p* < 0.01; ^***^ *p* < 0.001

**Table S5b.** Standardized results of the cross-lagged panel models: Covariates

|  | Model 1 - Identity and physical health perception | | | Model 2 - Identity and subjective well-being | | | Model 3 - Identity and psychological well-being | | | Model 4 - Identity and social well-being | | |
| --- | --- | --- | --- | --- | --- | --- | --- | --- | --- | --- | --- | --- |
| Covariates | T1🡪T2 | T1🡪T3 | T1🡪T4 | T1🡪T2 | T1🡪T3 | T1🡪T4 | T1🡪T2 | T1🡪T3 | T1🡪T4 | T1🡪T2 | T1🡪T3 | T1🡪T4 |
| Sex → Educational commitment | -.03 | -.01 | .00 | -.02 | -.00 | .01 | -.02 | .00 | .01 | -.01 | .00 | .02 |
| Sex → Educational in-depth exploration | .07^**^ | .03 | .02 | .07^**^ | .03 | .03 | .08^**^ | .04 | .03 | .08^***^ | .04 | .03 |
| Sex → Educational reconsideration of commitment | -.04 | -.05 | -.07^*^ | -.03 | -.05 | -.06^*^ | -.03 | -.04 | -.06^*^ | -.04 | -.05 | -.06^*^ |
| Sex → Interpersonal commitment | -.03 | -.06^*^ | .01 | -.02 | -.05 | .02 | -.01 | -.04 | .03 | -.02 | -04 | .02 |
| Sex → Interpersonal in-depth exploration | .07^**^ | .03 | .00 | .07^**^ | .03 | .01 | .09^***^ | .05 | .02 | .08^***^ | .04 | .01 |
| Sex → Interpersonal reconsideration of commitment | -.02 | -.10^***^ | -.02 | -.02 | -.09^**^ | -.01 | -.01 | -.09^**^ | -.01 | -.00 | -.08^**^ | -.00 |
| Sex → Physical health perception | -.04 | -.05^*^ | -.06^*^ |  |  |  |  |  |  |  |  |  |
| Sex → Subjective well-being |  |  |  | -.10^***^ | -.06^*^ | -.10^***^ |  |  |  |  |  |  |
| Sex → Psychological well-being |  |  |  |  |  |  | -.10^***^ | -.08^**^ | -.07^*^ |  |  |  |
| Sex → Social well-being |  |  |  |  |  |  |  |  |  | -.04^***^ | -.12^***^ | -.11^***^ |
| Age → Educational commitment | -.02 | .02 | -.06^*^ | -.02 | .02 | -.06^*^ | -.02 | .02 | -.07^*^ | -.02 | .02 | -.06^*^ |
| Age → Educational in-depth exploration | -.09^***^ | -.00 | -.02 | -.09^***^ | -.00 | -.02 | .09^***^ | -.00 | -.03 | .09^***^ | .00 | -.02 |
| Age → Educational reconsideration of commitment | .00 | .01 | .04 | .01 | .02 | .04 | .00 | .02 | .04 | .00 | .02 | -.02 |
| Age → Interpersonal commitment | .06^*^ | .02 | -.00 | .06^*^ | .02 | -.00 | .05^*^ | .02 | -.01 | .06^*^ | .02 | .04 |
| Age → Interpersonal in-depth exploration | -.04 | .01 | -.04 | .04 | .02 | -.03 | .09^***^ | .02 | -.03 | .04 | .02 | -.03 |
| Age → Interpersonal reconsideration of commitment | -.02 | -.03 | -.06 | -.02 | -.02 | -.05 | -.02 | -.02 | -.05 | -.02 | -.02 | -.05 |
| Age → Physical health perception | -.10^***^ | -.02 | -.04 |  |  |  |  |  |  |  |  |  |
| Age → Subjective well-being |  |  |  | -.01 | .02 | .01 |  |  |  |  |  |  |
| Age → Psychological well-being |  |  |  |  |  |  | -.01 | .02 | .02 |  |  |  |
| Age → Social well-being |  |  |  |  |  |  |  |  |  | -.02 | -.02 | -.00 |

*Notes*: T=Time; Sex: 0 = Boys, 1 = Girls; Age: 0 = First year, 1 = Third year.

^*^ *p* < 0.05; ^**^ *p* < 0.01; ^***^ *p* < 0.001

**Table S5c.** Standardized results of the cross-lagged panel models: Correlations and correlated changes

|  | Model 1 - Identity and physical health perception | | | | Model 2 - Identity and subjective well-being | | | | Model 3 - Identity and psychological well-being | | | | Model 4 - Identity and social well-being | | | |
| --- | --- | --- | --- | --- | --- | --- | --- | --- | --- | --- | --- | --- | --- | --- | --- | --- |
|  | T1 | T2 | T3 | T4 | T1 | T2 | T3 | T4 | T1 | T2 | T3 | T4 | T1 | T2 | T3 | T4 |
| Educational commitment ↔ Educational in-depth exploration | .58^***^ | .43^***^ | .48^***^ | .48^***^ | .58^***^ | .43^***^ | .47^***^ | .48^***^ | .58^***^ | .43^***^ | .47^***^ | .48^***^ | .58^***^ | .43^***^ | .47^***^ | .48^***^ |
| Educational commitment ↔ Educational reconsideration of commitment | -28^***^ | -.09^***^ | -.09^**^ | -.09^**^ | -28^***^ | -.09^**^ | -.09^**^ | -.09^**^ | -.28^***^ | -.09^**^ | -.09^**^ | -.10^***^ | -.28^***^ | -.09^***^ | -.09^**^ | -.09^**^ |
| Educational commitment ↔ Interpersonal commitment | .16^***^ | .14^***^ | .15^***^ | .15^***^ | .16^***^ | .14^***^ | .15^***^ | .15^***^ | -.16^***^ | .14^***^ | .15^***^ | .14^***^ | .16^***^ | .14^***^ | .15^***^ | .14^***^ |
| Educational commitment ↔ Interpersonal in-depth exploration | .03 | .12^***^ | .13^***^ | .13^***^ | .03 | .12^***^ | .13^***^ | .12^***^ | .03 | .12^***^ | .13^***^ | .12^***^ | .03 | .12^***^ | .13^***^ | .12^***^ |
| Educational commitment ↔ Interpersonal reconsideration of commitment | -.02 | .03 | .04 | .04^**^ | -.02 | .03 | .03 | .04 | -.02 | .03 | .03 | .03 | -.02 | .03 | .03 | .03 |
| Educational commitment ↔ Physical health perception | .16^***^ | .08^***^ | .08^***^ | .09^***^ |  |  |  |  |  |  |  |  |  |  |  |  |
| Educational commitment ↔ Subjective well-being |  |  |  |  | .30^***^ | .19^***^ | .20^***^ | .22^***^ |  |  |  |  |  |  |  |  |
| Educational commitment ↔ Psychological well-being |  |  |  |  |  |  |  |  | .31^***^ | .21^***^ | .21^***^ | .23^***^ |  |  |  |  |
| Educational commitment ↔ Social well-being |  |  |  |  |  |  |  |  |  |  |  |  | .27^***^ | .20^***^ | .21^***^ | .24^***^ |
| Educational in-depth exploration ↔ Educational reconsideration of commitment | -.01 | .10^***^ | .11^***^ | .11^***^ | -.01 | .10^***^ | .11^***^ | .11^***^ | -.01 | .10^***^ | .10^***^ | .11^***^ | -.01 | .10^***^ | .10^***^ | .11^***^ |
| Educational in-depth exploration ↔ Interpersonal Commitment | .07^*^ | .11^***^ | .12^***^ | .12^***^ | .07^*^ | .11^***^ | .12^***^ | .12^***^ | .07^*^ | .10^***^ | .11^***^ | .11^***^ | .07^*^ | .10^***^ | .11^***^ | .11^***^ |
| Educational in-depth exploration ↔ Interpersonal in-depth exploration | .15^***^ | .21^***^ | .22^***^ | .22^***^ | .15^***^ | .21^***^ | .22^***^ | .22^***^ | .15^***^ | .21^***^ | .22^***^ | .22^***^ | .15^***^ | .21^***^ | .22^***^ | .22^***^ |
| Educational in-depth exploration ↔ Interpersonal reconsideration of commitment | .10^**^ | .09^***^ | .09^***^ | .09^***^ | .10^*^ | .09^***^ | .09^***^ | .09^***^ | .10^*^ | .09^***^ | .09^***^ | .09^***^ | .10^**^ | .08^***^ | .09^***^ | .09^***^ |
| Educational in-depth exploration ↔ Physical health perception | .05 | .02 | .02 | .02 |  |  |  |  |  |  |  |  |  |  |  |  |
| Educational in-depth exploration ↔ Subjective well-being |  |  |  |  | .12^**^ | .07^***^ | .08^***^ | .08^***^ |  |  |  |  |  |  |  |  |
| Educational in-depth exploration ↔ Psychological well-being |  |  |  |  |  |  |  |  | .15^***^ | .11^***^ | .10^***^ | .12^***^ |  |  |  |  |
| Educational in-depth exploration ↔ Social well-being |  |  |  |  |  |  |  |  |  |  |  |  | .14^***^ | .11^***^ | .11^***^ | .13^***^ |
| Educational reconsideration of commitment ↔ Interpersonal commitment | -.04 | .06^**^ | .07^**^ | .07^**^ | -.04 | .06^**^ | .06^**^ | .067^**^ | -.04 | .06^**^ | .06^**^ | .06^**^ | -.04 | .06^**^ | .06^**^ | .06^**^ |
| Educational reconsideration of commitment ↔ Interpersonal in-depth exploration | .09^**^ | .11^***^ | .10^***^ | .10^***^ | .09^**^ | .11^***^ | .10^***^ | .10^***^ | .09^*^ | .10^***^ | .10^***^ | .10^***^ | .09^**^ | .11^***^ | .11^***^ | .10^***^ |
| Educational reconsideration of commitment ↔ Interpersonal reconsideration of commitment | .11^***^ | .12^***^ | .12^***^ | .13^***^ | .11^***^ | .13^***^ | .12^***^ | .13^***^ | .11^***^ | .13^***^ | .12^***^ | .13^***^ | .11^***^ | .13^***^ | .12^***^ | .13^***^ |
| Educational reconsideration of commitment ↔ Physical health perception | -.05 | -.02 | -.02 | -.02 |  |  |  |  |  |  |  |  |  |  |  |  |
| Educational reconsideration of commitment ↔ Subjective well-being |  |  |  |  | -.14^***^ | -.05* | -.05* | -.05* |  |  |  |  |  |  |  |  |
| Educational reconsideration of commitment ↔ Psychological well-being |  |  |  |  |  |  |  |  | -.10^**^ | -.01 | -.01 | -.00 |  |  |  |  |
| Educational reconsideration of commitment ↔ Social well-being |  |  |  |  |  |  |  |  |  |  |  |  | -.10^***^ | .02 | .02 | .02 |
| Interpersonal commitment ↔ Interpersonal in-depth exploration | .50^***^ | .44^***^ | .45^***^ | .43^***^ | .50^***^ | .44^***^ | .45^***^ | .43^***^ | .50^***^ | .44^***^ | .45^***^ | .42^***^ | .50^***^ | .44^***^ | .45^***^ | .43^***^ |
| Interpersonal commitment ↔ Interpersonal reconsideration of commitment | -.29^***^ | -.15^***^ | -.16^***^ | -.16^***^ | -.29^***^ | -.15^***^ | -.15^***^ | -.16^***^ | -.29^***^ | -.15^***^ | -.16^***^ | -.16^***^ | -.29^***^ | -.16^***^ | -.16^***^ | -.16^***^ |
| Interpersonal commitment ↔ Physical health perception | .14^***^ | .09^***^ | .09^***^ | .09^***^ |  |  |  |  |  |  |  |  |  |  |  |  |
| Intepersonal commitment ↔ Subjective well-being |  |  |  |  | .20^***^ | .15^***^ | .15^***^ | .16^***^ |  |  |  |  |  |  |  |  |
| Interpersonal commitment ↔ Psychological well-being |  |  |  |  |  |  |  |  | .33^***^ | .18^***^ | .17^***^ | .18^***^ |  |  |  |  |
| Interpersonal commitment ↔ Social well-being |  |  |  |  |  |  |  |  |  |  |  |  | .31^***^ | .17^***^ | .17^***^ | .19^***^ |
| Interpersonal in-depth exploration ↔ Interpersonal reconsideration of commitment | -.02 | .15^***^ | .14^***^ | .15^***^ | .00 | .15^***^ | .15^***^ | .15^***^ | -.00 | .15^***^ | .15^***^ | .15^***^ | .00 | .15^***^ | .15^***^ | .15^***^ |
| Interpersonal in-depth exploration ↔ Physical health perception | -.05 | .01 | .01 | .01 |  |  |  |  |  |  |  |  |  |  |  |  |
| Interpersonal in-depth exploration ↔ Subjective well-being |  |  |  |  | .02 | .05^*^ | .05^*^ | .05^*^ |  |  |  |  |  |  |  |  |
| Interpersonal in-depth exploration ↔ Psychological well-being |  |  |  |  |  |  |  |  | .12^***^ | .07^**^ | .07^**^ | .07^**^ |  |  |  |  |
| Interpersonal in-depth exploration ↔ Social well-being |  |  |  |  |  |  |  |  |  |  |  |  | .12^***^ | .09^***^ | .08^***^ | .10^***^ |
| Interpersonal reconsideration of commitment ↔ Physical health perception | -.06* | -.07^**^ | -.07^**^ | -.08^**^ |  |  |  |  |  |  |  |  |  |  |  |  |
| Interpersonal reconsideration of commitment ↔ Subjective well-being |  |  |  |  | -.11^***^ | -.02 | -.02 | -.02 |  |  |  |  |  |  |  |  |
| Interpersonal reconsideration of commitment ↔ Psychological well-being |  |  |  |  |  |  |  |  | -.12^***^ | -.04 | -.04 | -.04 |  |  |  |  |
| Interpersonal reconsideration of commitment ↔ Social well-being |  |  |  |  |  |  |  |  |  |  |  |  | -.04 | .05^*^ | .05^*^ | .06^*^ |

*Notes*: T=Time

^*^ *p* < 0.05; ^**^ *p* < 0.01; ^***^ *p* < 0.001

**Table S6.** Sensitivity analyses

|  | **Sex** | | | **Age** | | |
| --- | --- | --- | --- | --- | --- | --- |
|  | **Boys** | **Girls** | **Wald test** | **Younger adolescents** | **Older adolescents** | **Wald test** |
| **Model 1 – Identity and physical health perception** |  |  |  |  |  |  |
| Physical health perception ↔ Educational in-depth exploration (correlated changes) | .06^*^ | -.03^*^ | 5.24^*^ |  |  |  |
| **Model 2 – Identity and subjective well-being** |  |  |  |  |  |  |
| Educational reconsideration of commitment → Subjective well-being |  |  |  | -.04 | .05^*^ | 7.40^**^ |
| Subjective well-being ↔ Interpersonal reconsideration of commitment (baseline) |  |  |  | -.15^***^ | -.02 | 4.66^*^ |
| **Model 3 – Identity and psychological well-being** |  |  |  |  |  |  |
| Interpersonal in-depth exploration → Psychological well-being |  |  |  | -.07^**^ | .05 | 4.88^*^ |
| Psychological well-being → Educational reconsideration of commitment |  |  |  | -.00 | .08^**^ | 5.39^*^ |
| Psychological well-being → Interpersonal commitment |  |  |  | .05 | .15^***^ | 9.11^**^ |
| Psychological well-being → Interpersonal in-depth exploration |  |  |  | -.01 | .09^***^ | 5.68^*^ |
| Psychological well-being ↔ Interpersonal reconsideration of commitment (baseline) |  |  |  | -.17^***^ | -.05 | 3.86^*^ |
| **Model 4 – Identity and social well-being** |  |  |  |  |  |  |
| Interpersonal commitment → Social well-being | .03 | .12^***^ | 4.49^*^ |  |  |  |
| Social well-being → Educational commitment | .12^***^ | .02 | 7.35^**^ |  |  |  |
| Interpersonal in-depth exploration → Social well-being |  |  |  | -.07^**^ | .01 | 4.01^*^ |
| Social well-being → Interpersonal in-depth exploration |  |  |  | -.03 | .06^**^ | 8.31^**^ |

*Notes.* ^*^ *p* < 0.05; ^**^ *p* < 0.01; ^***^ *p* < 0.001
